# Redox–Oligomeric State of Peroxiredoxin-2 and Glyceraldehyde-3-Phosphate Dehydrogenase in Obstructive Sleep Apnea Red Blood Cells under Positive Airway Pressure Therapy

**DOI:** 10.3390/antiox9121184

**Published:** 2020-11-26

**Authors:** Cristina Valentim-Coelho, Fátima Vaz, Marília Antunes, Sofia Neves, Inês L. Martins, Hugo Osório, Amélia Feliciano, Paula Pinto, Cristina Bárbara, Deborah Penque

**Affiliations:** 1 Laboratório de Proteómica, Departamento de Genética Humana, Instituto Nacional de Saúde Dr. Ricardo Jorge, 1649-016 Lisboa, Portugal; cristina.valentim@insa.min-saude.pt (C.V.-C.); fatima.vaz@insa.min-saude.pt (F.V.); sofia.neves@insa.min-saude.pt (S.N.); ines.martins@insa.min-saude.pt (I.L.M.); 2 ToxOmics—Centre of Toxicogenomics and Human Health, Universidade Nova de Lisboa, 1150-082 Lisboa, Portugal; 3 Centro de Estatística e Aplicações da Universidade de Lisboa e Departamento de Estatística e Investigação Operacional, Faculdade de Ciências, Universidade de Lisboa, 1749-016 Lisboa, Portugal; marilia.antunes@ciencias.ulisboa.pt; 4 i3S–Instituto de Investigação e Inovação em Saúde, Universidade do Porto, 4200-135 Porto, Portugal; hosorio@i3s.up.pt; 5 Ipatimup–Institute of Molecular Pathology and Immunology of the University of Porto, University of Porto, 4200-135 Porto, Portugal; 6 Department of Pathology, Faculty of Medicine, University of Porto, 4200-319 Porto, Portugal; 7 Serviço de Pneumologia, Centro Hospitalar Lisboa Norte—CHLN, 1649-035 Lisboa, Portugal; af.pp@clix.pt (A.F.); paulagpinto@gmail.com (P.P.); cristina.barbara@chln.min-saude.pt (C.B.); 8 Instituto de Saúde Ambiental—ISAMB, Faculdade de Medicina, Universidade de Lisboa, 1649-026 Lisboa, Portugal

**Keywords:** obstructive sleep apnea (OSA), positive airway pressure (PAP), peroxiredoxin-2 (PRDX2), glyceraldehyde-3-phosphate dehydrogenase (GAPDH), Cys-sulfinylation/Cys-sulfonylation, proteomics-biomarkers

## Abstract

In this study, we examined the effect of six months of positive airway pressure (PAP) therapy on Obstructive Sleep Apnea (OSA) red blood cell (RBC) proteome by two dimensional difference gel electrophoresis (2D-DIGE) - based proteomics followed by Western blotting (WB) validation. The discovered dysregulated proteins/proteoforms are associated with cell death, H_2_O_2_ catabolic/metabolic process, stress response, and protein oligomerization. Validation by nonreducing WB was performed for peroxiredoxin-2 (PRDX2) and glyceraldehyde-3-phosphate dehydrogenase (GAPDH) by using antibodies against the sulfinylated/sulfonylated cysteine of these proteins to better evaluate their redox–oligomeric states under OSA and/or in response to PAP therapy. The results indicated that the redox–oligomeric state of GAPDH and PRDX2 involving overoxidation by sulfinic/sulfonic acids were differentially modulated in OSA RBC, which might be compromising RBC homeostasis. PAP therapy by restoring this modulation induced a higher oligomerization of overoxidized GAPDH and PRDX2 in some patients that could be associated with eryptosis and the chaperone “gain” of function, respectively. This varied response following PAP may result from the complex interplay between OSA and OSA metabolic comorbidity. Hence, information on the redox status of PRDX2 and GAPDH in RBC will help to better recognize OSA subtypes and predict the therapeutic response in these patients. GAPDH monomer combined with body mass index (BMI) and PRDX2 S-S dimer combined with homeostatic model assessment for insulin resistance (HOMA-IR) showed to be very promising biomarkers to predict OSA and OSA severity, respectively.

## 1. Introduction

Obstructive Sleep Apnea (OSA) has emerged as a major public health issue with a high proportion of socio-economic burden. OSA is characterized by repetitive upper airway apnea/hypopnea during sleep leading to intermittent hypoxemia, sleep fragmentation, and consequently homeostasis perturbation [1,2,3]. Non-treated OSA can result in cognitive and behavioral deficits, cancer, cardiovascular and metabolic disorders, and early mortality [1,2,3]. More than 200 million people worldwide may be affected by OSA, but the majority (60–90%) remains undiagnosed and thus untreated [4]. The gold-standard diagnosis of OSA, the overnight lab-based polysomnography (PSG) is expensive, cumbersome, and not widely available [1,2,3]. Diagnosis with home-monitoring equipment is an alternative not without costs, nor is it easily accessible, and confirmation by lab-PSG is often necessary [5]. The effective treatment for OSA is the nasal (continuous) positive airway pressure (PAP) [6], although negative results have been also reported, and not all patients benefit from it [7].

For all these reasons, a cost-efficient blood biomarker-based tool for OSA screening in a large population and/or to identify individuals at risk for developing complications from OSA constitute an extreme societal need [2,8]. Biomarkers able to predict or monitor the effectiveness of PAP treatments will also advance OSA clinical care [2,8,9].

Our group has investigated for the first time to our knowledge the red blood cell (RBC) proteome from OSA patients to better understand the underlying mechanisms while uncovering potential biomarkers for a more cost-effective OSA diagnosis or as predictors of treatment [2,10,11]. Why RBCs? RBCs are the most abundant cells in the body. Dysfunction in RBC homeostasis has been described as a potential source of systemic inflammation that leads to metabolic and cardiovascular diseases such as those associated with OSA [12]. By two dimensional difference gel electrophoresis (2D-DIGE) proteomics, we demonstrated that OSA induces differential changes in RBC cytoplasmic proteome [10,11], in which redox-regulators such as peroxiredoxin-2 (PRDX2) are the most dysregulated. Since RBC is devoid of any translational machinery, these changes might result from post-translational modifications (PTM) regulation. Western blot (WB) validation using non-reducing SDS-PAGE indicated that different redox–oligoforms of PRDX2 correlated with OSA severity and OSA metabolic status [10,11]. Six months of PAP treatment decreased monomeric/dimeric sulfinic–sulfonic (overoxidized) forms of PRDX2 in OSA RBC while it increased sulfinic–sulfonic decameric forms of this protein, which are described as having a chaperone protective function [10].

Herein, we intended to better investigate the effect of six months of PAP treatment on the OSA RBC proteome by using the 2D-DIGE proteomic approach followed by WB validation for the most relevant differential proteins. The results indicated that PAP induces significant concerted modulation in RBC redox regulators such as in glyceraldehyde-3-phosphate dehydrogenase (GAPDH) and PRDX2. In non-treated patients, the RBC expression level of these proteins was shown to be associated with OSA risk or OSA disease severity, respectively.

## 2. Materials and Methods

### 2.1. Patients and Samples

One hundred four consecutive male subjects with clinically suspected OSA syndrome were recruited under informed consent for blood biobanking collection and clinical and biochemical evaluation as we previously described [10]. Exclusion criteria were female gender (to avoid hormonal influence), shift workers, other sleep disorders, neuromuscular disease, heart failure, diabetes, neoplasia, acute disease, and previous PAP treatment. PAP therapy was prescribed for patients diagnosed with OSA as we previously described [13,14]. For proteomics studies, blood samples were fractionated into plasma, buffy-coat, and RBC aliquots before −80 °C storage until use. Biochemical analysis were performed according to the hospital standard procedures and include 24 h urinary catecholamines, glycemic and lipidic profiles, cardiovascular marker (homocysteine), and complete hemogram at time zero (t0, hospitalization day for lab-PSG diagnosis) and time six (t6; after six months of PAP treatment).

Distinct RBC sample sets were selected from the biobank for the discovery phase and validation phase. For the discovery phase, RBC samples from subjects with primary snoring (RDI ≤ 5/h; *n* = 10) and severe OSA before and after six months of PAP treatment (RDI ≥ 30/h; *n* = 10/group) were selected (Cohort I, Table 1). For the validation phase, RBC samples from snorer subjects (RDI ≤ 5/h; *n* = 23) and OSA patients with mild (RDI ≥ 5/h, but ≤ 15/h; *n* = 16) or moderate to severe OSA (RDI ≥ 15/h; *n* = 20) that underwent six months of PAP treatment were selected to validate GAPDH as described below (Cohort II, Table 2, total *n* = 56). From this Cohort II, the same controls (*n* = 18) and patient samples (*n* = 19) in a total of 37 samples were selected to validate PRDX2 (Cohort III, Table S1, Supplementary Materials).

The protocol of this project was approved by the Ethics Committees of the Centro Hospitalar Lisboa Norte (CHLN), Hospital Santa Maria, Lisboa, Portugal (6 January 2012) and Instituto Nacional de Saúde Dr. Ricardo Jorge, Lisboa, Portugal (9 April 2013). The project was registered at the Comissão Nacional de Proteção de Dados (CNPD) and all patients gave written informed consent.

### 2.2. Discovery Phase: 2D-DIGE Proteomics

RBC samples (Cohort I) were lysated by incubation with 5 mM phosphate buffer pH 7.4 (1:20) containing 1:100 cocktail of protease inhibitors (P8340, Sigma Aldrich, Darmstadt, Germany) for 30 min at 4 °C followed sonication 10 s/40 amplitude/pulse mode (Ultrasonic Processor, VibraCell, Sonics & Materials Inc, Newtown, CT, USA). After centrifugation at 25,000× *g* for 30 min at 4 °C (Centrifuge 5417, Eppendorf, Hamburg, Germany), the supernatants were recovered for further hemoglobin (Hb) depletion using Hemovoid depletion columns (Biotech Support Group, Monmouth JCT, NJ, USA), according to the manufacture’s protocol. The obtained Hb-depleted fractions were concentrated and buffer-exchanged with 25 mM NH_4_HCO_3_ pH 8.4 by centrifugal filtration using 3-kD Molecular Weight Cut-Offs (MWCO) (Amicon Ultra 4, Millipore, Darmstadt, Germany) spin concentrators. Protein concentration was determined by a colorimetric assay (Pierce^TM^ 660 nm Protein Assay Kit, Thermo Fisher Scientific, Waltham, MA, USA) according to the manufacturer’s protocol. Samples were stored at −80 °C until use.

Samples were analyzed individually by 2D-DIGE mini-gel (7 cm IPG strips) using the CyDye DIGE fluor minimal dyes Cy3 and Cy5 from GE Healthcare, Chicago, USA. Briefly, samples (10 µg/sample) were dried in speed vacuum and resuspended in 1.25 µL of lysis buffer (7 M urea (Amersham Biosciences, Little Chalfont, UK), 2 M thiourea (Merck, Darmstadt, Germany), 2% (*w*/*v*) 3-[(3-cholamidopropyl)dimethylammonio]-1-propanesulfonate (CHAPS, Sigma Aldrich, Darmstadt, Germany), Tris 30 mM (Sigma Aldrich, Darmstadt, Germany) and incubated with 400 pmol Cy5 solution for 30 min at 4 ºC in the dark. The labeling reaction was stopped by adding 1 µL of 10 mM lysine, and samples were incubated for another 10 min at 4 °C in the dark. For internal standard (IS), a sample from each subject was pooled together and labeled with Cy3 as above described. Each Cy5 labeled sample was combined with an equal amount of Cy3 labeled IS (10 µg:10 µg) and mixed with lysis buffer with a trace of bromophenol blue (Merck, Darmstadt, Germany) to 125 µL final volume and let to solubilize during 1 h 30 at 22 °C (with occasional vortex). To ensure an optimal protein focusing, ampholyte pH 3–10 Non Linear (NL) (Serva, Heidelberg, Germany) was added to 1% final concentration before loading the samples on to 7 cm Immobilized pH gradient (IPG) strips pH gradient of 3–10 NL (non-linear) (GE Healthcare, Chicago, USA) previously rehydrated for 20 h at RT with 112 µL of lysis buffer. IEF was performed in an Ettan IPGphor 3 (GE Healthcare, Chicago, IL, USA) in ceramic manifold with cup loading of the sample and focused as follows: step and hold 300 V for 30 min, gradient 1000 V for 30 min, gradient 5000 V for 1 h 30 min, step and hold 5000 V 1 h followed by step and hold at 10 V for 10 min. The maximum current per strip was set to 50 µA.

Prior, 2nd dimension stripes were equilibrated once with 3 mL of SDS equilibration buffer (6 M urea, 75 mM Tris-HCL pH 8.8, 29.3% glycerol (86%) (Sigma Aldrich, Darmstadt, Germany), 2% SDS (Sigma Aldrich, Darmstadt, Germany) and 0.002% of bromophenol blue] including 1% of DTT (Sigma Aldrich, Darmstadt, Germany) (15 min, RT) to accomplish the reduction of disulfide bonds, which was followed by the derivatization of cysteine residues blocking buffer containing 4% of iodacetamide (Sigma Aldrich, Darmstadt, Germany) (15 min, RT).

Second-dimension separation was performed using XCell SureLock Mini-Cell (Invitrogen by Thermo Fisher Scientific, Waltham, MA, USA) using NuPAGE 4–12% Bis-tris ZOOM Gel, 1.0mm IPG well (Invitrogen by Thermo Fisher Scientific, Waltham, USA) running 125 V, 2 h, and RT.

Gels were scanned at 100 µm resolution using an Amersham Biosciences Typhoon 8400, variable imager to obtain IS (Cy3) image and sample (Cy5) image as we previously described [11,15]. Spot detection, gel matching, and statistical analysis were performed by the Progenesis SameSpots, version 4.5 (Nonlinear Dynamics, UK). Abundance values of matched spots across all mini gel images, expressed as normalized volume, were compared between conditions, so that each spot could be assigned a score of relative significant difference in terms of *p*-value (<0.05). The relative content alteration of each spot across the study conditions was expressed by fold change values, which were calculated by the ratio of the mean normalized volumes of a certain spot in each condition. Spots decreasing their abundance were represented by negative fold values, which were calculated as the inverse of the previous ratio multiplied by −1.

### 2.3. Protein Identification by Mass Spectrometry

A preparative 2D-mini gel (7 cm IPG strip, GE Healthcare, Chicago, IL, USA) stained Coomassie (Brilliant Blue G, Sigma Aldrich, Darmstadt, Germany), containing an equal amount of each non-labeled individual sample mixed with 10 µg of IS labeled sample, in a total of 75 µg of protein, was performed for further spot cut-off for mass spectrometry (MS) protein identification. The introduction of some labeled sample into preparative gel facilitates gel match with analytical gels for spot location and picking for MS analysis. The protein spot of interest was cut off from the gel and trypsin digested, as we previously described [16,17]. Tryptic peptides, suspended in 50% (*v*/*v*) ACN and 0.1% (*v*/*v*) trifluoroacetic acid (TFA, for HPLC, ≥99.0%, Sigma Aldrich, Darmstadt, Germany) and submitted to sonication in an ultrasonic bath during 15 min, were directly applied on a 100-well matrix-assisted laser desorption/ionization (MALDI) plate with 5 mg/mL α-cyano-4-nydroxycinnamic acid (α-CHCA, 1:1) prepared in 0.1% TFA/60% ACN (Merck, Darmstadt, Germany) (*v*/*v*) and allowed to co-crystallize at RT. Peptides were analysed on an Applied Biosystems SCIEX 4800 Plus MALDI Proteomics Analyser with time-of-flight/time-of-flight (TOF/TOF) ion optics exactly as described [18]. The identified proteins are shown in Table S2 (Supplementary Materials).

### 2.4. Protein Annotation and Classification

Protein annotation properties were acquired using the Database for Annotation, Visualization and Integrated Discovery (DAVID) v6.8 (Frederick, MD, USA) [19]. This open-source tool retrieves a set of biological and functional information such as GO terms, subcellular location, molecular function, and association with biological process and/or disease with *p*-values of over-representation ≤0.05.

### 2.5. Validation Phase: Western Blotting Analysis

The validation of proteomics data was performed by WB for two proteins GAPDH and PRDX2 on RBC samples from Cohort II (Table 2) and Cohort III (Table S1), respectively. RBC samples were 1:100 diluted with 100 mM of *N*-ethylmaleimide (NEM, crystalline, ≥98% HPLC, Sigma Aldrich, Darmstadt, Germany) in PBS buffer for 10 min at 4 °C to prevent exogenous-induced oxidation. Samples (500 µL) were lysed in 1:1 double distilled (dd) H_2_O with protease inhibitors cocktail 1:100 (P8340, Sigma Aldrich, Darmstadt, Germany), centrifuged at 9279× *g* for 10 min at 4 °C (Centrifuge 5417, Eppendorf, Hamburg, Germany), and the cytoplasmic supernatants were recovered for protein quantification as above described.

To keep protein disulfide bonds formation in an oligomerization state, samples were separated (70 ug/lane) by non-reducing 4–12% SDS-PAGE mini gels followed by WB analysis using 1:2000 rabbit polyclonal anti-GAPDH antibody (ab9485, abcam, Cambridge, UK), 1:500 mouse monoclonal anti-GAPDH-SO_3_ antibody (4A1) (Abfrontier, South Korea), 1:20,000 rabbit anti-PRDX2 antibody (ab59539, abcam, Cambridge, UK), or 1:3000 rabbit anti-PRDX-SO_2/3_ antibody (ab16830, abcam, Cambridge, UK) to investigate the different redox oligomeric states of GAPDH and PRDX2, respectively, as described [20,21].

### 2.6. Statistical Analysis

Descriptive analyses for clinical and analytical data were expressed as mean ± standard deviation (SD), and frequency (% values) was used to characterize the groups. One-way ANOVA (analysis of variance) was used to compare statistically more than two groups; Student-*t* test was used to compare Snorer and OSA groups. A paired Student-*t* test was used to evaluate the effect of (before/after) PAP treatment in an OSA group set. A correlation of variables was carried out using the Pearson correlation test. The level of statistical significance was set at 5% (*p*-value < 0.05). The above-mentioned statistical analysis was performed using IBM SPSS Statistics 25 (Armonk, NY, USA).

Subsequent statistical analysis was performed using R (version 3.6.1) (Vienna, Austria) and RStudio (version 1.2.5019) (Boston, MA, USA).

Logistic regression models and subsequent ROC curves were built to evaluate the discriminative power of the different redox–oligomeric forms of GAPDH or PRDX2 and the ability to predict OSA severity (mild–moderate vs. severe). Cut-off points were chosen to maximize sensitivity and specificity. Logistic regression was performed using the function glm from base R. ROC curve analysis was done using the R package pROC.

Spaghetti plots (Spaghetti.Plot function from R package CorrMixed) were used to represent the evolution of the variables under analysis from t0 to t6. The Generalized Estimating Equation (GEE) was used to estimate logistic regression models with the time/group interaction term to assess the differences of the impact of PAP in the subgroups under comparison [22]. Logistic GEE models were fitted using the function geeglm from R package geepack. Radar Plots (using R package fmsb) were built to illustrate graphically the differences between the average profiles of groups. Radar charts allow representing simultaneously a small group (preferably 3 to 4) of profiles composed by several quantitative variables. Values for each variable are scaled to the interval [0,1], with zero corresponding to the minimum observed value and one corresponding to the maximum observed value. Intermediate values are scaled proportionally. The Gower similarity measure [23], which is computed as one minus the Gower dissimilarity measure, was chosen to quantify the similarity of the average profile of the groups, since it is the similarity measure that best resembles the rationale under the radar plot. To compute the Gower dissimilarity measure, each variable is first standardized by dividing each entry by the range of the corresponding variable after subtracting the minimum value; the rescaled variables have a range of [0,1], exactly. In the presence of quantitative variables only, the Gower dissimilarity between two profiles is equal to the average of the absolute value of the differences for each component of the profile vectors. Values of the Gower similarity measure range from zero to one, with one indicating total similarity and zero indicating the least possible similarity. The Gower dissimilarity measure was computed using the daisy function from the R package cluster.

## 3. Results

### 3.1. Patients: Clinical, Biochemical, and Metabolic Characteristics

The results are summarized in Table 1 and Table 2 (and Table S1). In both Cohort I (Table 1, Discovery phase) and Cohort II (Table 2, Validation phase), significant differences between OSA and Snorers patients were observed regarding the PSG parameters as expected but also the body mass index (BMI), abdominal perimeter, and insulin resistance, determined by homeostatic model assessment of insulin resistance (HOMA-IR), which were higher in those with OSA (*p* < 0.05 Student *t*-test). No significant differences were observed for the other parameters, except that in Cohort II, there were significantly higher insulin levels in OSA patients compared to Snorers (Student *t*-test, *p* < 0.05) was observed (Table 2).

After six months of PAP treatment (compliance with mean usage > 4 h night), patients reported a significant decrease in excessive daytime somnolence, as evaluated by the Epworth Sleepiness Scale (EPW) score; the hemogram data, although showing clinical normal reference values, revealed a small, but significant, decrease in the RBCs as well as platelets count, hemoglobin concentration, and hematocrit in patients after PAP treatment (paired Student *t*-test, *p* < 0.05) (Table 2). There were no significant changes in glucose and lipid profile and cardiovascular marker after treatment (Table 2).

### 3.2. PAP Treatment Induces Changes in the RBC Proteome

**Figure 1:**
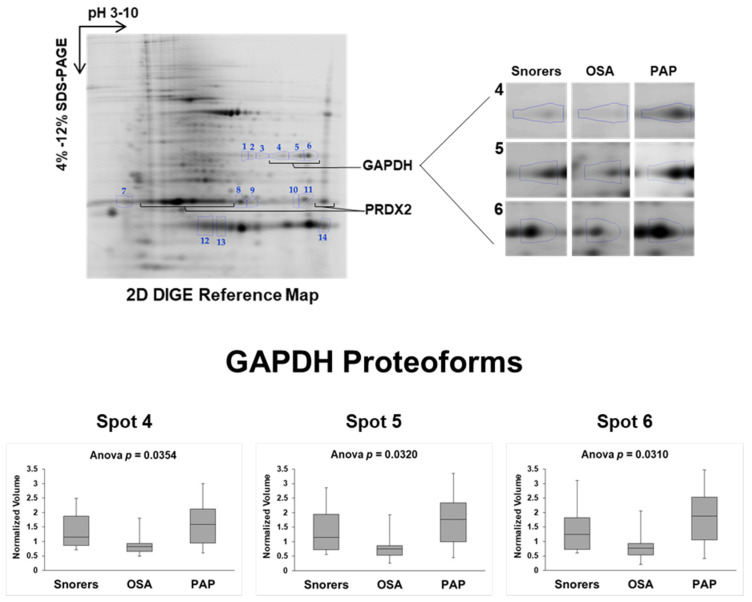
Two dimensional difference gel electrophoresis (2D-DIGE) reference map of hemoglobin-depleted red blood cells (RBCs) from Snorers subjects and Obstructive Sleep Apnea (OSA) patients before and after positive airway pressure (PAP) treatment. Fourteen protein/proteoforms spots differentially abundant (fold change ≥ 1.2; Anova *p* < 0.05) among Snorer RBC and OSA RBC before and after six months of PAP treatment are numbered and indicated with circles on the reference mini 2D-DIGE map image displayed. The MS characterization of these proteins is fully described in Figure 2 and Table S2, Supplementary Materials. Three proteoforms of glyceraldehyde-3-phosphate dehydrogenase (GAPDH), corresponding to spots 4–6 on the 2D-DIGE, were identified significantly decreased in OSA RBC that after six months of PAP treatment increased to the Snorer levels as shown by the respective graphical representations of spot normalized volume profile among the analyzed groups (shown at the bottom of the figure). Graphical representation of all identified spot-proteins as differentially abundant among the groups under study is shown in Figure S1 (see Supplementary Materials).

**Figure 2:**
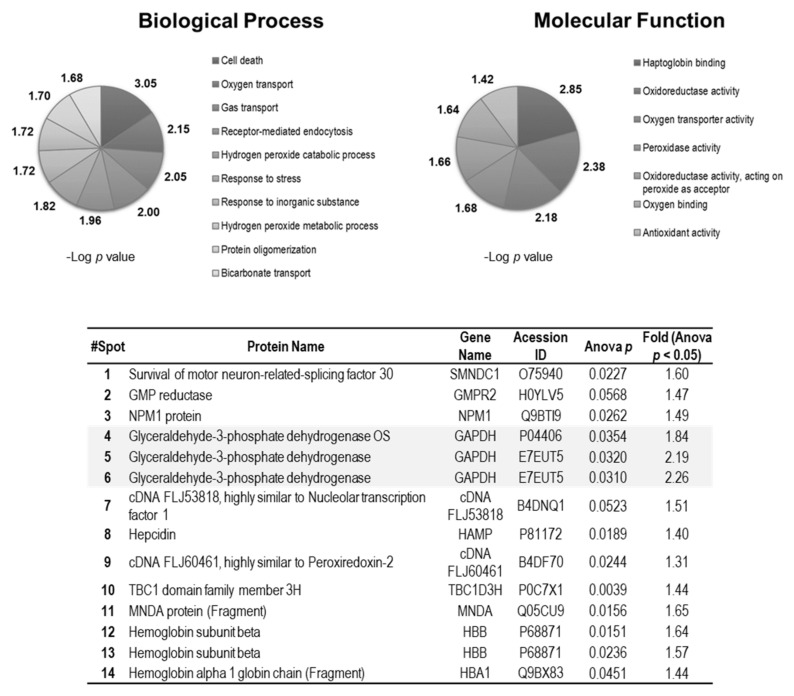
Biological processes and molecular functions of the most significantly changed proteins among Snorers and OSA before and after PAP treatment. Table at the bottom shows the list of proteins/proteoforms identified differentially abundant among Snorers and OSA before and after six month of PAP treatment (fold change ≥ 1.2; Anova *p* < 0.05). The biological processes and molecular functions associated with these proteins acquired from Database for Annotation, Visualization and Integrated Discovery (DAVID) v6.8 are shown at the top of the figure.

A total of 85 protein spots were visualized on 2D-DIGE mini-gel maps, of which 14 were differentially abundant (fold change ≥ 1.2; Anova *p* < 0.05) among Snorers and OSA before and after PAP treatment, as shown in Figure 1. From these differentially abundant spots, 12 were identified by MS corresponding to unique proteins, suggesting the existence of post-translational modification (PTM) regulations in these proteins (Figure 2). The most evident were three GAPDH proteoforms that were identified as significantly reduced in OSA RBC compared to the ones observed in Snorer RBC. However, after PAP, the abundance of these GAPDH proteoforms increased in these patients, reverting to levels comparable to those of Snorers (Figure 1). Among other proteins, proteoforms for Peroxiroxin-2 (PRDX2) were also identified (Figure 2, Table S2). Changes in the acidic PRDX2 proteoform abundance was also observed among the groups, although it was not statistically significant. 

### 3.3. GAPDH and PRDX2 Redox–Oligoforms in OSA before and after PAP Treatment

**Figure 3:**
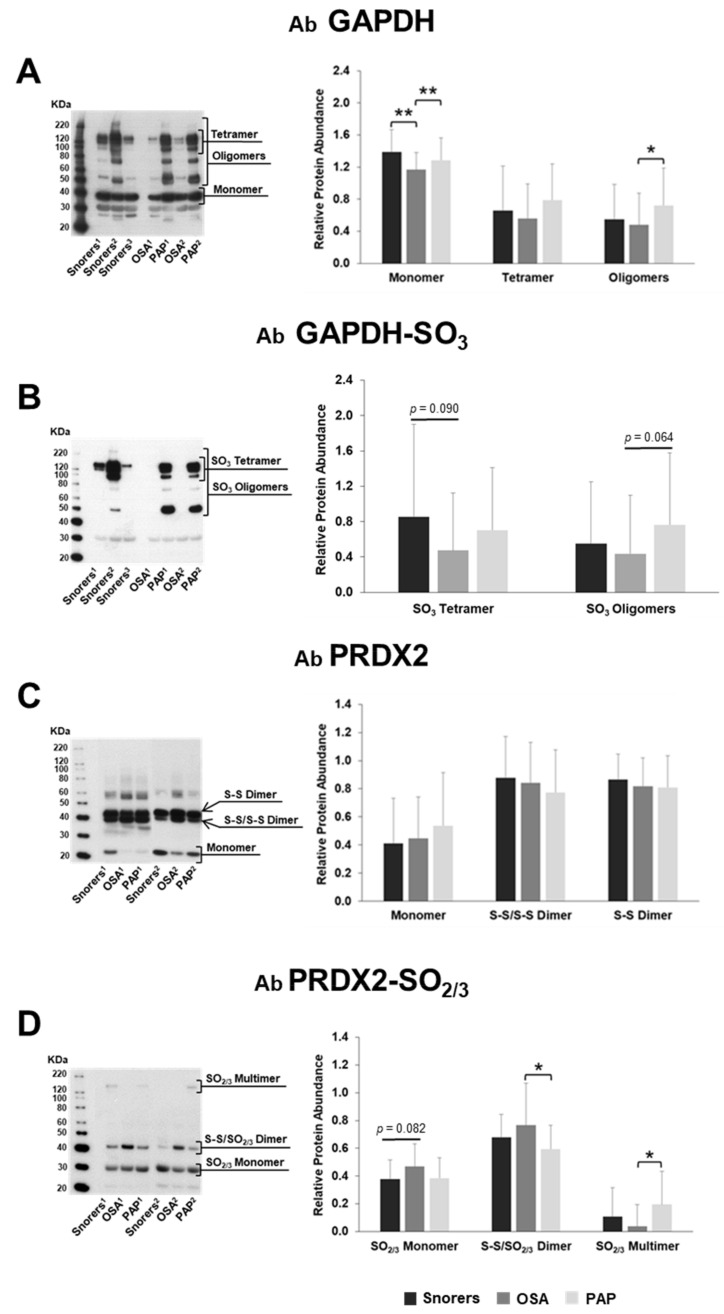
Western blot (WB) validation of the proteomics data showing changes in GAPDH and PRDX2 redox/oligomeric states in RBC isolated from Snorers and OSA patients before or after PAP treatment. On the left, representative non-reducing SDS-PAGE WB fluorograms of RBC proteins immunoreactive to the antibody (Ab) GAPDH (**A**), Ab GAPDH-SO_3_ (**B**); Ab PRDX2 (**C**) or Ab PRDX2-SO_2/3_ (**D**). On the right, the respective graphic plots of GAPDH (**A**,**B**) and PRDX2 (**C**,**D**) redox/oligomeric form relative normalized protein abundances calculated by densitometric analysis (see Material and Methods). Statistically significant differences (*p* < 0.05) and (*p* < 0.01) between groups are indicated (*) and (**), respectively.

GAPDH and PRDX2 were selected for further WB validation of the proteomics data using Cohort II (Table 2) and a sub-cohort of Cohort II (Cohort III, Table S1), respectively. Knowing that GAPDH function as a homotetrameric struc ture can be regulated by different PTMs, including cysteine overoxidation by sulfinylation-sulfonylation (SO_2_/SO_3_) [24] and the functional dimer–decameric transition of PRDX2 is also mainly regulated by cysteine oxidation (disulfide bond) or cysteine overoxidation (SO_2/3_) [21,25], a non-reducing SDS-PAGE was used to keep as much as possible the redox–oligomeric states of these proteins during the electrophoresis separation. WB was performed by using specific antibodies against GAPDH, PRDX2, GAPDH-SO_3_, or PRDX2-SO_2/3_ proteoforms. The results are shown in Figure 3A–D.

In addition to the monomeric form (37 kDa), several oligoforms of GAPDH from 50 to 200 kDa, which includes the tetrameric form (≈150 kDa), were also detected (Figure 3A). The abundance of these oligoforms, specifically the abundance of GAPDH monomer (37 kDa), was shown to be significantly decreased in OSA RBC compared to Snorer RBC. After PAP treatment, the abundance of this monomeric form significantly increased in OSA RBC to the levels comparable to the ones observed in Snorer RBC (Figure 3A). These results corroborate the 2D-DIGE proteomic data (Figure 1). All these GAPDH oligoforms but not the monomeric ones were recognized by the antibody for SO_3_ GAPDH, indicating the presence of sufinylation–sulfonylation oxidation modification in these oligoforms (Figure 3B). PAP treatment seemed to induce the increase of oxidation modification in GAPDH tetrameric or oligomeric forms but with *p*-value not sufficient to reach statistical significance (Figure 3B).

As we previously confirmed, in non-reducing SDS-PAGE condition, the dimers or multimers of PRDX2 in cells that are fully reduced or fully overoxidized were denatured and visualized as ≈21 kDa monomers by WB using either Ab-PRDX2 or Ab-PRDX2-SO_2/3_ (Figure 3C,D). In contrast, using Ab-PRDX2 dimeric/multimeric oxidized forms that have two disulfide-linked (S-S/S-S) peroxidatic cysteines or one disulfide linked, whereas the other cysteine is either reduced or overoxidized (S-S/SH or S-S/SO_2/3_) were denatured and visualized as ≈50 kDa (denominated here as S-S/S-S dimer) and 52 kDa (S-S dimer), respectively [10,26]. Multimers (or decamers) fully or partially disulfide-linked (≈200 kDa) that are not denatured were hardly observed as predicted by [21] (Figure 3C). By using the Ab-PRDX2-SO_2/3_ on RBC lysates analyzed by a non-reducing SDS-PAGE condition, monomer/dimer/multimer of PRDX2 that contain sulfinylated/sulfonylated (SO_2/3_) cysteine were exclusively detected as described [27] (Figure 3D).

A tendency for an increase of PRDX2 monomer level—in particular, the overoxidized (SO_2/3_) monomer—was observed in OSA RBC compared to Snorer ones (*p* = 0.82), which confirmed the proteomics data. A significant increase in S-S/SO_2/3_ dimeric forms of PRDX2 was also observed associated with OSA (Figure 3D). After PAP, these overoxidized forms were decreased with statistical significance (*p* < 0.05) for dimeric forms (Figure 3D). Moreover, multimeric overoxidized (SO_2/3_) forms of PRDX2 were also detected mostly exclusively in OSA patients after six months of PAP treatment. PRDX2-SO_2/3_ multimeric forms were also detected in some Snorer subjects (Figure 3D) and in one of 36 OSA patients analyzed before treatment.

### 3.4. GAPDH and PRDX2 Correlation before and after PAP

The correlation between the relative abundance of the different redox/oligomeric states of GAPDH and PRDX2 evaluated by WB in the OSA cohort (*n* = 19) before and after PAP treatment was studied. The significant results (*p* < 0.05*, *p* < 0.001***) are shown in Table 3. Before PAP treatment, PRDX2 disulfide form (S-S/S-S), which was described as associated with the peroxidase catalytic cycle of the protein, was significantly negatively correlated with the GAPDH tetramer (*r* = −0.512*) and GAPDH SO_3_ tetramer/oligomer (*r* = −0.483*/ *r* = −0.473*). After PAP, the overoxidized monomeric forms of PRDX2 negatively correlated with the GAPDH monomer (*r* = −0.551*), GAPDH tetramer/oligomer (*r* = −0.551*/ *r* = −0.485*), and GAPDH SO_3_ tetramer/oligomer (*r* = −0.506*/*r* = −0.516*). The PRDX2 SO_2/3_ multimer that mostly was detected after treatment was positively correlated with the GAPDH monomer (*r* = 0.526*) and strongly positively correlated with the GAPDH tetramer/oligomer (*r* = 0.777***/*r* = 0.712***) and GAPDH SO_3_ tetramer/oligomer (*r* = 0.838***/*r* = 0.787***).

### 3.5. Correlation between GAPDH and PRDX2 with OSA Clinical Parameters before and after PAP

The correlation between the relative abundance of each different redox/oligomeric state form of GAPDH and PRDX2 detected by WB and the clinical and biochemical parameters measured in OSA patients before and after PAP treatment were studied. The statistically significant results (*p* < 0.05*, *p* < 0.01**, *p* < 0.001***) are shown in Table 4.

Before treatment, GAPDH monomers correlated positively with the PSG RDI parameter (*r* = 0.375*) and RBC (*r* = 0.389*) and Hb (*r* = 0.392*) hematological parameters. Significant negative correlations between the GAPDH oligomer (*r* = −0.337*) and overoxidized GAPDH tetramer/oligomer (*r* = −0.359*/ *r* = −0.354*) with the HbA1C metabolic parameter were observed before treatment. The PRDX2 monomer showed a strong positive correlation with TG (*r* = −0.593**) and positive correlation with HCY (*r* = 0.469*). All dimeric forms of PRDX2 (S-S/S-S, S-S/SO_2/3_, S-S dimer) correlated positively with PLT (*r* = 0.510*, r = 0.552*, *r* = 0.508*) and negatively with RDW (*r* = −0.577**, −0.457*, −0.465*) hematological parameters. PRDX2 S-S dimer showed also a significant negative correlation with the PSG RDI parameter (*r* = −0.570*) and with INS (*r* = −0.462*) and HOMA-IR (*r* = −0.476*) metabolic parameters.

After PAP treatment, the GAPDH monomer positively correlated with RDW (*r* = 0.363*). Positive correlations between the GAPDH tetramer (*r* = 0.336*) and GAPDH SO_3_ tetramer/oligomer (*r* = 0.421*/ *r* = 0.362*) with the HbA1C metabolic parameter were observed after treatment. The GAPDH SO_3_ tetramer also positively correlated with the TG (*r* = 0.341*) and HCY (*r* = 0.355*) parameters at this time. The GAPDH tetrameric/oligomeric forms, including the overoxidized ones, strongly positively correlated with ADR (*r* = 0.490**/ *r* = 0.436**; *r* = 0.553***/ *r* = 0.479**) after treatment. The overoxidized (SO_2/3_) monomeric forms of PRDX2 strongly negatively correlated with GLC (*r* = −0.601**) and ADR (*r* = −0.456*) parameters. The PRDX2 S-S/SO_2/3_ dimer positively correlated with HbA1C (*r* = 0.549*), and the PRDX2 SO_2/3_ multimer positively correlated with TG (*r* = 0.479 *) and strongly with ADR (*r* = 0.772***) after treatment.

### 3.6. GAPDH and PRDX2 Logistic Regression Models and ROC Curve Analysis for Predicting OSA or OSA Severity

**Figure 4:**
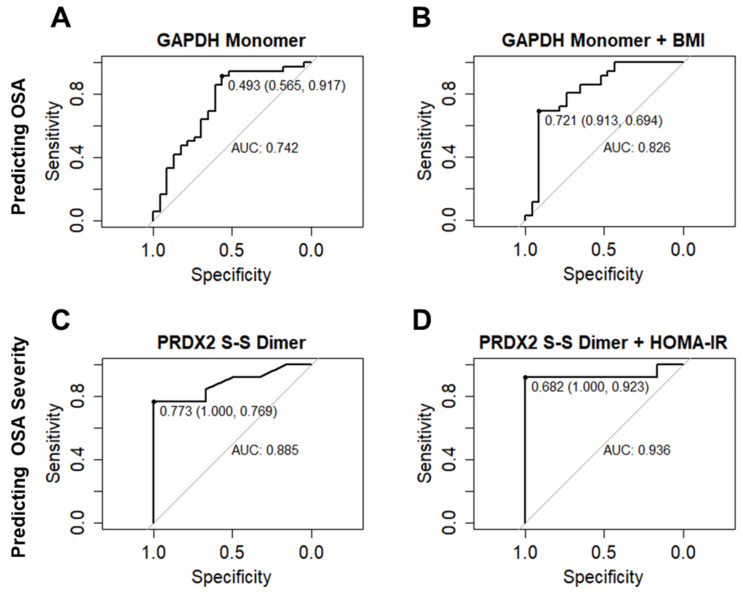
Receiver Operating Characteristic (ROC) curves for the models using GAPDH and PRDX2 to classify patients at risk for OSA or OSA severity. The different redox–oligomeric forms of GAPDH or PRDX2 were used as predictors in logistic regression models and Receiver Operating Characteristic (ROC) curves were used to assess their potential discriminative power in classifying patients at risk for OSA or as predictor of OSA severity. The best results to predict patients at risk for OSA were obtained for the GAPDH monomer alone (AUC: 0.742; 0.917 sensitivity; and 0.565 specificity) (**A**) or combined with Body Mass Index (BMI) parameter (AUC: 0.826; 0.694 sensitivity; and 0.913 specificity) (**B**). The best results to discriminate severe OSA from mild–moderate OSA were obtained for the PRDX2 S-S Dimer alone (AUC: 0.885; 0.769 sensitivity; and 1.000 specificity) (**C**) or combined with the homeostatic model assessment for insulin resistance (HOMA-IR) metabolic parameter (AUC: 0.936; 0.923 sensitivity; and 1.000 specificity) (**D**). The respective optimal cut-off values and the specificity and sensitivity values (within parenthesis) are displayed in the plots.

Following logistic regression models, ROC curves were used to compare the discriminative power of the different redox–oligomeric forms of GAPDH or PRDX2 to classify patients at risk for OSA or as predictors of OSA severity. The most significant results of ROC analysis are shown in Figure 4. The GAPDH monomer presented the highest performance to predict patients at risk for OSA with an AUC value of 0.742 (sensitivity: 0.917; specificity: 0.565) (Figure 4A). The combination of the GAPDH monomer with the clinical parameter BMI slightly improved this performance by presenting an AUC value of 0.826 (sensitivity: 0.694; specificity: 0.913) (Figure 4B).

To discriminate severe OSA patients from mild–moderate OSA patients, the S-S PRDX2 dimer presented the best ROC AUC of 0.885 (sensitivity: 0.769; specificity: 1) (Figure 4C). However, the model considering a combination of the S-S PRDX2 dimer with the HOMA-IR parameter presented the greatest ROC AUC of 0.936 (sensitivity: 0.923; specificity: 1) for this OSA severity discrimination (Figure 4D).

### 3.7. Clinical Response in PAP-Induced PRDX2 SO_2/3_ Multimer

After PAP treatment, the SO_2/3_ multimeric form of PRDX2 was identified in eight (42%) of 19 evaluated OSA patients as highly and strongly positively correlated with the level of GAPDH multimers/oligomers over- or not overoxidized (Table 3).

In attempt to understand whether the tendency for an occurrence of PRDX2 SO_2/3_ multimer after six months of treatment is associated with a specific clinical state before treatment or with a specific clinical response after treatment, patients were split into two subgroups, OSA with or without PAP-induced PRDX2 SO_2/3_ multimer at time t6. Then, these subgroups were statistically compared for the clinical parameters measured at before (t0) and after treatment (t6) by using Logistic GEE regression models with the interaction term time:group. These classes of models allow accounting for the dependence induced by the multiple (two) measurements while evaluating the effect of the treatment and the possible differences between groups both at t0 and t6. These models allow assessing whether the groups under comparison were similar or not at t0 as well as to quantifying and comparing the variation in both groups from t0 to t6, which could possibly be attributed or associated with the treatment.

**Figure 5:**
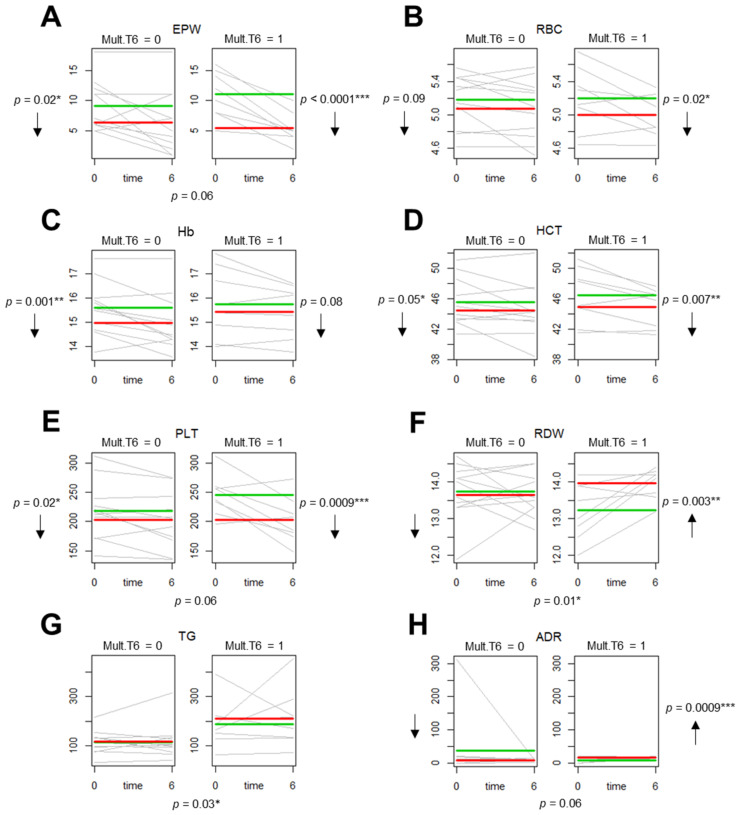
Spaghetti plot displaying clinical parameter data evaluated in two subgroups of OSA patients before (t0) and after six months of PAP treatment (t6). The OSA subgroup with (pred.mult = 1) and without (pred.mut = 0) induced PRDX2 SO_2/3_ multimer after six months of PAP treatment were compared by evaluating the clinical parameters under study at time t0 and t6, using Logistic GEE regression models. The most significant data were obtained for EPW (**A**), RBC (**B**), Hb (**C**), HCT (**D**), PLT (**E**), RDW (**F**), TG (**G**) and ADR (**H**) clinical parameters as shown. Green line indicates the average value of a clinical parameter at t0, and the red line indicates the average value of this clinical parameter at time t6.

Figure 5 shows the most significant results graphically displayed by spaghetti plots. Lines in green represent the fitted value of a clinical parameter at t0, whereas lines in red represent the fitted values for this clinical parameter in t6. Models showed that there were significant differences between t0 and t6 in both subgroups for the EPW and hematological parameters RBC, Hb, HCT, platelets and RDW (Figure 5A–F). Although the decreased of EPW, RBC, HTC, and PLT, at t6 (red line) compared to t0 (green line) was shown to have a higher statistical significance in the OSA subgroup with the PAP-induced PRDX2 SO_2/3_ multimer (Mult.T6 = 1) than in the OSA subgroup without this multimer induction (Mult.T6 = 0) (Figure 5A–E); these decreases were considered statistically similar between these two OSA subgroups except that the decreased of EPW and PLT were borderline statistically different (*p* = 0.06) between the subgroups (Figure 5A,E). In contrast, RDW significantly increased in OSA with the PAP-induced PRDX2 SO_2/3_ multimer (Mult.T6 = 1) (*p* = 0.003 **) at t6 (red line) compared with t0 (green line), and this increase was statistically significant between the subgroups (*p* = 0.014 *). In fact, in OSA without this multimer (Mult.T6 = 0), RDW presented a tendency to decrease after treatment (t6) although with no statistical significance (*p* = 0.12) (Figure 5F). No significant differences were observed in the lipidic parameters between the subgroups, except that before treatment (t0), the triglycerides (TG) showed a highly significant increased (*p* = 0.03 *) in the OSA subgroup with PAP-induced PRDX2 SO_2/3_ multimer (Mult.T6 = 1) compared with OSA without this multimer (Mult.T6 = 0) at this time (t0) (Figure 5G). There were no significant differences in catecholamines between the subgroups except that after treatment, adrenaline (ADR) significantly increased (*p* = 0.0009 ***) in OSA with PAP-induced PRDX2 SO_2/3_ multimer (Mult.T6 = 1), and this modulation was borderline significant (*p* = 0.06) between the two subgroups (Figure 5H).

**Figure 6:**
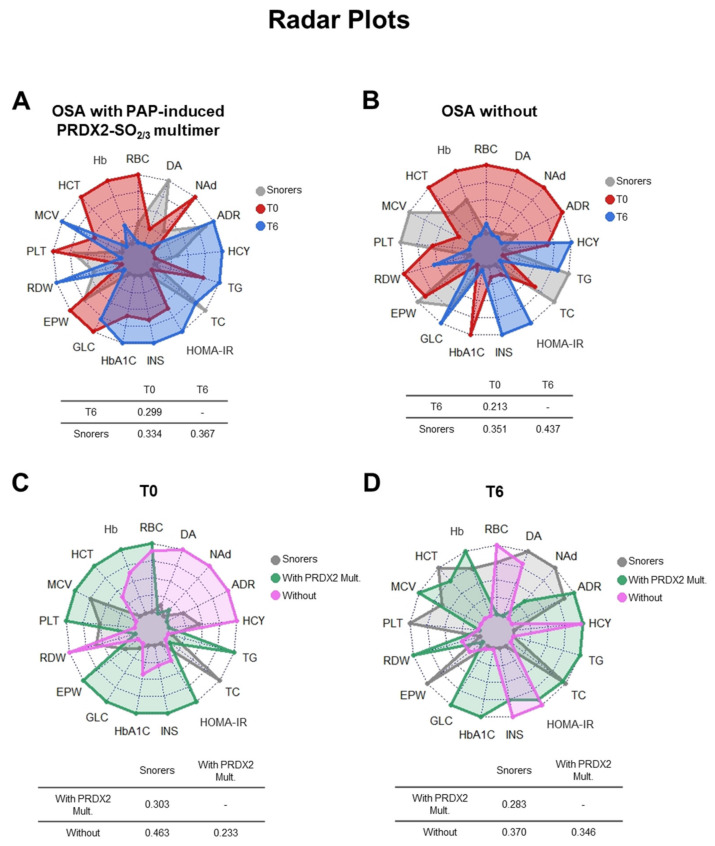
The study clinical parameters are displayed in one dimension comparing the OSA subgroup with (**A**) or without PAP-induced PRDX2-SO_2/3_ response (**B**), measured at time t0 (T0, red) and t6 (T6, blue) with the Snorer group as a control reference (gray); or OSA subgroup with (green) and without PAP-induced PRDX2-SO_2/3_ response (rose) at t0 (**C**) and t6 (**D**) with the Snorer group as a control reference (gray). Each clinical parameter is represented on a different radius of a radar plot. The centroid points in the periphery represented the highest average value, whereas the centroid points in proximity to the center corresponded to the lowest average value for a clinical parameter. The similarities between the different groups at different conditions are measured by the Gower similarity measure (the closer to 1, the more similar the groups) and the results indicated at the bottom to each corresponding radar plot.

Radar plots followed by Gower’s similarity measure calculation allow comparing the average profile of groups considering a collection of variables of interest. Figure 6 shows Radar plots displaying in one dimension the study of the clinical parameters comparing the Snorer and OSA subgroups with (A) or without PAP-induced PRDX2 SO_2/3_ response (B) at time t0 and t6 and the Snorer and both OSA subgroups with and without PAP-induced PRDX2 SO_2/3_ response at time t0 (D) or at time t6 (E). Each of the clinical parameters under study is represented on a different radius of a radar plot. The length of the radius was proportional to the index value. The centroid points in the periphery (radius equal to one) represented the highest average value, whereas the centroid points in proximity to the center corresponded to the lowest average value for a clinical parameter.

Intermediate values are plotted proportionally, considering the relative position to the extremes. To evaluate the (dis)similarity among Snorer and OSA subgroups before and after PAP, the Gower distance was calculated to obtain the similarity matrix, as explained in the Material and Methods section. The closer to the value 1, the greater the similarity between the two groups. The results indicated that according to the clinical parameters under study, both OSA subgroups, i.e., with and without PAP-induced PRDX2 SO_2/3_ multimer, seemed to be quite different from the Snorer group at time t0 (before PAP) (Gower similarity measure of 0.334 and 0.351, respectively) and this difference tended to diminish after treatment (t6) (Gower similarity measure slightly increased to 0.367 and 0.437, respectively) (Figure 6A,B). Interestingly, at time t0, these OSA subgroups were more dissimilar to each other (Gower similarity measure of 0.233) than they were with the Snorer group (Gower similarity measure of 0.303 and 0.463, respectively) (Figure 6C). After treatment (t6), the OSA subgroup with PAP-induced PRDX2 SO_2/3_ response showed to be more distant (dissimilar) to the Snorer group (Gower similarity measure of 0.283) than the OSA subgroup without this multimer (Gower similarity measure of 0.37). The difference between these OSA subgroups seemed to diminish after treatment (Gower similarity measure of 0.346).

## 4. Discussion

In this study, we investigated the effect of six months of PAP treatment on the OSA RBC proteome by 2D-DIGE-mini gel-based proteomics followed by WB validation. The results indicated that RBC proteins/proteoforms associated with the biological process such as cell death, H_2_O_2_ catabolic/metabolic process, stress response, and protein oligomerization were the most relevant and significantly changed among Snorers as control and OSA patients before and after treatment. One of these proteins, the redox regulators PRDX2 and GAPDH, were further validated in an independent cohort of patients by nonreducing WB using antibodies (Ab) against cysteine (Cys) sulfinylated/sulfonylated (SO_2/3_) of either PRDX2 or GAPDH to better evaluated the redox–oligoform behavior of these proteins in OSA RBC or OSA RBC under PAP. 

PRDX2 is a member of six thiol peroxidases playing a key redox–oligomeric regulated role in the antioxidant defense, redox signaling, and chaperone function [21]. Previously, we observed a significant increase of overoxidized (SO_2/3_) PRDX2 monomer/dimer in OSA RBC that, after PAP treatment, decreased with the increase of PRDX2 SO_2/3_ multimeric forms reported, as associated with chaperone function [10]. In this study carried out in a larger number of patients, the level of PRDX2 SO_2/3_ monomer was increased in OSA compared with Snorers, although with a borderline significance (*p* = 0.082). However, we confirmed that PAP treatment induced a significant decrease in the level of low molecular overoxidized forms of PRDX2, especially PRDX2 SO_2/3_ dimers, whereas a significant induction of PRDX2 SO_2/3_ multimer occurred. Interestingly, this PAP-induced PRDX2 SO_2/3_ multimer phenomenon was strongly positively correlated with PAP-induced GAPDH, especially GAPDH SO_3_ tetramer (r = 0.838, *p* < 0.0001 ***).

### 4.1. RBC GAPDH as Predictor of OSA

GAPDH is a ubiquitous glycolytic enzyme with multiple alternative and unrelated moonlighting functions driving by the cellular and environmental context [24,28]. GAPDH is a recognized key redox-sensor regulated by oxidative modifications induced by hydrogen peroxide (H_2_O_2_) and other oxidants generated either from metabolism or stress conditions. The multifunctional diversity of GAPDH is regulated by its oligomerization, post-translational modifications (PTMs), and subcellular localization. Cytoplasmic GAPDH functions as a tetramer protein (≈150 kDa) composed of four identical 37 kDa subunits, each with a single catalytic cysteine (Cys 152) thiol sensitive group [24,28]. In human serum, secreted GAPDH has been described as a multimer consisting of high-molecular weight subunits besides the 37 kDa subunit [29]. Deficiencies in GAPDH activity or expression have been implicated in diseases such as neurodegenerative diseases, cardiovascular disorders, diabetes, and cancer [30,31]. Herein, we demonstrated for the first time our knowledge the association between the RBC GAPDH redox–oligomeric state and OSA and OSA response to PAP treatment. In OSA RBC, the general level of GAPDH, especially the monomeric forms, showed a significant decrease compared to the Snorer ones used as controls. Six months of PAP treatment showed to revert that by increasing the GAPDH monomer abundance in OSA RBC to similar levels detected in Snorer RBC. This PAP-induced GAPDH monomer is followed by an increase of GAPDH tetrameric/oligomeric forms, including sulfonylated (SO_3_) ones.

The oxidation of GAPDH active Cys is one of the key mechanistic events in regulating metabolic adaptation upon exposure to physiological or pathological pro-oxidant environments [28,32]. The oxidation of GAPDH catalytic Cys-152 inactivates the enzyme oxidoreductase activity, leading to metabolic reroute from glycolysis to the pentose phosphate pathway (PPP) to fulfill the acute demand for reducing equivalents (e.g., NADPH), which is crucial for the antioxidant defense system [33,34]. 

In RBC, the glucose metabolic route varies with O_2_ gradients along the normal course of RBC circulation. Under the high O_2_ partial pressure of the lung, RBC metabolism shifts toward PPP to generate NADPH to preserve the glutathione antioxidant homeostasis. Under the low O_2_ pressure of the peripheral tissues, where RBC must be distorted to pass through capillaries, RBC promotes glycolysis to generate ATP to compensate for the cation leaks induced by this mechanical stress and to promote further oxygen release and tissue oxygenation, thus relieving hypoxia. This metabolic shift alternation is modulated via competition between the glycolytic enzyme complex (constituted by > 60% of GAPDH) and deoxyHb for membrane band-3 binding [35]. Upon deoxygenation, this glycolytic enzyme complex, mainly GAPDH is displaced by deoxyHb to be actively redistributed into the cytoplasm to increase glycolytic flux. However, such redox-based regulatory mechanism is compromised by chronic exposure to hypoxia or oxidative stress, leading to pathological consequences. Hypoxia favors glycolysis and downregulates PPP, which impairs the capacities of RBC to cope with oxidative stress and ultimately its ability to carrying oxygen to tissues [36,37]. A study performed in high-altitude polycythemia (HAPC) subjects, a typical model of hypoxia-induced excessive erythrocytosis (increased RBC production to maintain the oxygen level in the body), demonstrated that the PPP metabolic pathway was decreased in these subjects compared to controls but increased after reoxygenation recovery at plain altitude, along with a significant decrease in the RBC and Hb level [38]. OSA is characterized by intermittent hypoxemia/reoxygenation events leading to oxidative stress [39]. The limited antioxidant capacity and greater systemic oxidative stress that are experienced by patients with severe OSA have been suggested as associated with a reduced glutathione level in RBC [40], whose recycling is PPP-dependent [41]. PAP therapy eliminates apnea/hypopnea events and stabilizes normal oxygen saturation and consequently diminishes oxidative-stress in patients [42]. OSA rarely leads to erythropoiesis [43]. In this study, although OSA hematological parameters were normal and not significantly different from Snores, PAP treatment decreased significantly the level of RBC, HCT, and Hb in these patients, confirming ours [14] and other previous studies [44,45]. In fact, one year of PAP therapy can cause anemia in some patients [46]. Along with that, PAP induced the GAPDH level content in RBC, including monomer and overoxidized tetrameric/oligomeric forms. Unfortunately, we did not evaluate whether the regulation of glycolysis/PPP metabolic ratio is impaired in OSA RBC due to OSA intermittent hypoxia nor the effect of PAP on that. However, our results with others’ evidence led us to speculate that OSA intermittent hypoxia might induce a PPP/glycolysis ratio imbalance in RBC, leading to low capacity to generate NADPH, which is a key reducing equivalent for glutathione recycling. Hypoxia is reported to up or down-regulate GAPDH mRNA and protein according to the cell type context [47]. Therefore, the observed decreased of GAPDH in OSA RBC could be an erythrocytic progenitor cell response to OSA intermittent hypoxia that is still visible in mature RBC. Down-regulating the GAPDH glycolytic enzyme in these patients’ cells could help minimize hypoxia-induced glycolysis over flux during RBC circulation in the lung. Interestingly, the GAPDH monomer content in OSA RBC positively correlated with OSA severity (RDI). This makes us speculate that patients with severe OSA, by not reducing GAPDH in RBC under severe intermittent hypoxia, have limited capacity to counteract the hypoxia-induced glycolytic reroute over PPP flux, which may explain the reported lower oxidative stress resistance in these patients [40].

These results let us investigate the putative value of each or combined redox–oligoforms of GAPDH for the prediction of OSA or OSA severity. The best ROC analysis data indicated that the GAPDH monomer with a cut-off point of 0.493 relative protein abundance has a specificity of 0.565 and sensitivity of 0.917 (AUC: 0.742) to predict OSA. However, the combination of GAPDH monomer with BMI, an easily measurable clinical parameter, improved the GAPDH monomer predictive ability for OSA screening (cut-off: 0.721 with 0.934 specificity and 0.694 sensitivity, AUC: 0.826). For OSA severity prediction, the combination of GAPDH monomer with HOMA-IR showed a good predictive value, cut-off: 0.720 with 1.000 specificity and 0.824 sensitivity, AUC: 0.895 (data not shown). However, the PRDX2 S-S dimer combined with HOMA-IR showed an even better predictive value for OSA severity (see the discussion below). Obesity and diabetes are highly prevalent in OSA [1], which justifies these data. Taken together, these results suggest that the GAPDH monomer in RBC, combined with BMI or HOMA-IR, are promising for OSA screening and OSA disease monitoring, respectively.

### 4.2. PAP-Induced Sulfonylated GAPDH Tetramer/Oligomer in RBC May Be Related with GAPDH Peroxidase Activity and/or Eryptosis Associated “Gain of Function”

As discussed above, PAP-induced oxygen saturation normalization might have a significant beneficial impact on OSA RBC metabolic physiology, since PAP was showed to normalize the GAPDH level while increasing the oxidized GAPDH content that is associated with PPP metabolic reroute to protect cells against oxidative stress. The catalytic Cys-SOH to Cys-SO_3_H transition in the subpopulations of GAPDH coincide with oxidoreductase activity inactivation and a gain in peroxidase activity, which enables GAPDH to enzymatically reduce H_2_O_2_ to water [32,48]. However, once sulfonylated GAPDH is formed, it is generally considered as an irreversible inactive form associated with protein misfolding prone to degradation [48]. Very recently, Lia et al., by determining the first crystal structure of GAPDH tetramer carrying sulfonylated modification in active Cys, showed that this modification induces an irreversible inactivation of the enzyme without causing significant structural changes on the tetrameric enzyme [49]. However, the exact “gain of function” associated with this overoxidation remains to be elucidated.

Sulfonylation derived from S-nitrosylation has been reported in GAPDH and associated with protein translocation to subcellular domains where it does not normally occur to stimulate a “gain of function” associated with apoptosis [50]. Under stress condition or normoxia recovery from hypoxia, erythrocytes undergo eryptosis, which resembles apoptosis in nucleated cells [51]. One of the common features among cell-death programs is the phosphatidylserine externalization, which is a signal for a quick cell removal by specialized phagocytes [51]. Interestingly, GAPDH is a phosphatidylserine binding protein involved in membrane fusion, which is a process associated with apoptosis [28,52]. However, the GAPDH redox/oligo state-based mechanism modulating this process remains to be determined. The increased RBC protein glycation, including glycated hemoglobin (HbA1C) induces phosphatidylserine externalization and thus RBC aging and eryptosis [53]. After PAP, a significant positive correlation between HbA1C and the tetrameric/oligomeric forms of GAPDH, especially the sulfonylated ones, was observed. This evidence associated with the fact that the levels of RBC and HTC decreased significantly after PAP treatment leads us to speculate that the induction of sulfonylated forms of GAPDH in response to PAP may be associated with the induction of programmed cell death in RBC, especially those exposed to a high level of HbA1c. An increase of RBC destruction causes greater size heterogeneity and thus a higher RDW [54]. Studies have reported either no change [55] or an increase in RDW after PAP treatment [44]. In this study, no significant change in RDW after PAP was observed. However, when we considered the two subgroups of OSA patients, with and without the occurrence of PRDX2 SO_2/3_ multimer after treatment (see below), the latter presented a significant increase in RDW after PAP along with a higher significant decrease in RBC and HTC compared with the OSA subgroup without the PAP-induced PRDX2 SO_2/3_ multimer. The GAPDH SO_3_ tetramer strongly significantly correlated with the occurrence of the PRDX2 SO_2/3_ multimer, which again strongly supports the idea that sulfonylation of the GAPDH tetramer in OSA RBC after PAP reoxygenation might be associated with a higher decrease of RBC and HTC level. In contrast, the occurrence of PRDX2 SO_2/3_ multimers in RBC may play a protective role in order to attenuate the PAP- induced RBC GAPDH SO_3_ multimer associated with eryptosis (see discussion below).

### 4.3. RBC PRDX2 as a Predictor of OSA Severity

As a typical 2-Cys peroxiredoxin, PRDX2 functions as a local regulator of ROS/RNO concentration and sensor and transducer of ROS/RNO signaling [21,56]. The minimal catalytic unit of 2-Cys PRDXs is dimeric, and it is associated into oligomeric structures, normally decamers, in which the reactivity of peroxidatic Cys as its susceptibility to overoxidation are increased. The peroxidatic Cys of each subunit reacts with H_2_O_2_ and is oxidized to sulfenic acid derivatives (-SOH) that form a disulfide bond (-S-S-) with the resolving Cys of another subunit. Disulfide dimeric form is subsequently reduced by the thioredoxin/NADPH system to enable a further peroxidase catalytic cycle of H_2_O_2_. Occasionally and with increasing H_2_O_2_ concentration, 2-Cys PRDXs can be overoxidized to sulfinic (-SO_2_H), which can still be reduced by sulfiredoxin or end up irreversibly overoxidized to sulfonic acids (-SO_3_H). This subsequent overoxidation causes the formation of high molecular weight oligomeric structures on 2-Cys PRDXs with “gain of” chaperone function to protects cells against stress-induced protein unfolding or aggregation [57]. In addition, overoxidation confers function on PRDXs associated with H_2_O_2_ signaling and circadian rhythm regulation [58]. In RBC, PRDX2 exhibits an overoxidation circadian rhythm as a result of ROS generated by Hb autooxidation, which occurs naturally in RBC [27]. Interestingly, PPP regulates the transcription and redox circadian rhythm of many genes/proteins including PRDX2 via NADPH metabolism. Thus, PPP inhibition has a significant impact on the normal circadian rhythm of these genes/proteins [59]. Hypoxia inhibit PPP [60], and hypoxia in OSA has been associated with circadian rhythm disruption [61,62]. We previously demonstrated that although RBC is devoid of transcriptional/translational machinery, day–night changes in proteins such as PRDX2 resulting from redox–oligo state modulation take place but were significantly different in OSA compared to Snorer [10]. It is plausible to assume that OSA hypoxia leads to significant changes in the circadian redox rhythm of RBC proteome, which includes PRDX2. Herein, by studying the effect of PAP treatment on OSA RBC proteome in the morning, when the most modulation may occur, we confirmed that in OSA, there is differential modulation in the overoxidation state of PRDX2 monomer and dimer, which is reversed by the treatment, along with the appearance of multimeric overoxidized forms. Oxidation induced decamer dissociation into dimers to facilitate reduction by the thioredoxin/NADPH system [63]. In contrast, overoxidation stabilizes PRDX2 decamers and de-couples the thioredoxin/NADPH system from PRDX reduction, which increases the availability of thioredoxin and NADPH to reduce other proteins or peptides such as glutathione under stress conditions. Sulfinylation involves the sulfenylation of both peroxidatic Cys in a dimer and the prior formation of disulfide bonds between the PRDX monomer partners [57]. Dimers with both peroxidatic Cys linked by disulfide bounds (S-S/S-S Dimer) undergo a peroxidase activity cycle, whereas dimers with both peroxidatic Cys overoxidized engage in a non-peroxidase “gain of function” cycle associated with oligomer modulate signaling and chaperone function [21]. By nonreducing WB using Ab-PRDXSO_2/3_, fully overoxidized decamer/dimers are dissociated and identified as SO_2/3_ monomers. However, dimers partially overoxidized and disulfide-linked (S-S Dimer or S-S/SO_3_ Dimer) can also be detected by Ab-PRDX2 and Ab-PRDXSO_2/3_ respectively, migrating at the same MW but higher than S-S/S-S Dimer. The functional significance of these dimers has yet to be established. Although overoxidation inactivates PRDX peroxidase activity, recent studies have suggested that overoxidized PRDX may retain some catalytic peroxide-removing activity under stress conditions [57], which may explain the identification of S-S dimer and S-S/SO_2/3_ dimer. In this study, S-S/S-S dimer but also S-S dimer and S-S/SO_2/3_ dimer correlated significantly and negatively with RDW and positively with PLT in nontreated OSA, suggesting that RBC and PLT homeostasis under OSA condition may turn up the peroxidase activity of PRDX2 in RBC. In nontreated OSA, the S-S dimer also correlated significantly and negatively with RDI, INS, and HOMA-IR metabolic parameters, and positively with EPW. Ab-PRDX2 identifies partially disulfide-linked dimers (S-S dimer) without discriminating the concomitant overoxidation. In addition to sulfinylation/sulfonylation, glutathionylation may also occur in PRDX2 peroxidatic Cys, leading to decamer dissociation into dimer without chaperone function [64]. Unfortunately, we have not investigated whether glutathionylation in PRDX2 would explain the correlation between S-S dimer and OSA severity (RDI). However, by evaluating the putative predicted value of individual or combined PRDX2 redox–oligoforms for OSA diagnosis or OSA severity, we observed that PRDX2 S-S dimer alone or combined with HOMA-IR showed the best ROC curve data able to discriminate severe OSA from mild–moderate OSA. PRDX2 S-S dimer alone with a cut-off point of 0.773 relative protein abundance has a 0.769 sensitivity and 1.000 specificity (AUC: 0.885), which was much improved when combined with the HOMA-IR metabolic parameter, showing a cut-off of 0.682 relative protein abundance with a 0.923 sensitivity and 1.000 specificity (AUC: 0.936) to predict OSA severity. These data strongly suggest that PRDX2, namely S-S dimer, is a promising prognostic biomarker in OSA.

### 4.4. PRDX2 SO_2/3_ Multimer May Protect RBC from PAP-Mediated Eryptosis

The occurrence of PRDX2 SO_2/3_ multimers in the RBC of OSA patients was observed almost exclusively after six months of PAP treatment and in about 45% of patients. No significant difference in compliance with PAP treatment that could explain the occurrence of this phenomenon in some patients but not in others was observed.

By using a logistic GEE regression model displayed by Spaghetti plots, we tried to assess whether there were differences between these two subgroups of OSA patients, i.e., with and without PAP-induced PRDX2 SO_2/3_ multimer, regarding the clinical parameters under study measured before and after treatment.

Before treatment, no significant differences were observed between these subgroups except for the TG parameter, which was significantly higher in the subgroup with PAP-induced PRDX2 SO_2/3_ multimer compared to the subgroup without this induction. No significant change in TG level was observed in both subgroups after treatment. However, the levels of both PRDX2 SO_2/3_ multimers and GAPDH SO_3_ tetramers were significantly positively correlated with plasma TG level after PAP. After treatment, the subgroup with PRDX2-SO_2/3_ multimer occurrence and thus with a higher increase of GAPDH SO_3_ tetramer/oligomer presented the most significant decreased in RBC and HTC along with the most significant increase in RDW, which suggest a higher eryptosis process in this patient’s subgroup. The PLT level was also greatly decreased in the subgroup with PAP-induced PRDX2 SO_3_ multimer. A high level of plasma TG is associated with both RBC aggregation [65] and platelet activation and aggregation [66]. Increased RBC aggregation and platelet activation have been observed in OSA patients [67,68]. The constant lipid exchange between plasma lipoproteins and RBC may explain aggregation in RBC by altering RBC membrane composition [65]. Therefore, RBC may be more sensitive to PAP-induced eryptosis in conditions where the TG is increased.

As above discussed, the PAP-induced GAPDH SO_3_ tetramer/oligomer may be related to PPP metabolic reroute but also with increased eryptosis after treatment, but what will be the role of PRDX2 SO_2/3_ multimer in this process? The overoxidation of PRDX2 by sulfinic acid promotes stable oligoforms and interaction with signaling or chaperone/misfolded proteins [57]. The overexpression of PRDX2 protected cells against oxidative-mediated apoptosis; conversely, PRDX2 knockdown exaggerated the cell death induced by multiple stimuli [69]. In RBC, PRDX2 may prevent hemolytic anemia from oxidative stress by stabilizing Hb [70]. The decameric form of PRDX2 can bind to Hb and protect Hb from oxidative-induced denaturation and aggregation in human and mouse [70]. Interestingly, the subgroup of patients with PAP-induced PRDX2 SO_2/3_ multimer, although showing a greater decrease in RBC and HCT, presented no significant decrease in Hb parameter after treatment. In contrast, the subgroup without PAP-induced PRDX2 SO_2/3_ multimer showed a higher significant decrease in Hb parameter but no significant decrease in RBC and HCT after PAP. One year of PAP therapy was reported to induce anemia in some OSA patients [46], which makes us speculate that the occurrence of PRDX2 SO_2/3_ multimer after PAP may protect patients at a risk for exaggerated GAPDH SO_3_ tetramer associated eryptosis such as those patients with high plasma TG and/or glycated Hb.

Certain catecholamines, which includes ADR, are thought to suppress eryptosis [51]. In this study, urinary ADR significantly and positively correlated with PRDX2 SO_2/3_ multimer and GAPDH SO_3_ tetramer after PAP. After treatment, urinary ADR greatly increased in the subgroup of patients with PAP-induced PRDX2 SO_2/3_ multimer, whereas no significant change in ADR was observed in the subgroup without this induction. A possible mechanistic interaction between overoxidized PRDX2/GAPDH and ADR induction to protect RBCs from undergoing eryptosis under certain physiological condition remain to be elucidate.

The varied PRDX2/GAPDH redox–oligomeric responses following PAP treatment may result from a balance of interacting pathophysiologic features of OSA and OSA metabolic comorbidity. Radar plots displaying in one dimension all measured clinical parameters followed by Gower’s similarity measure calculation allowed us to evaluate the (dis)similarity among Snorer and the OSA subgroups, i.e., with/without PAP-induced PRDX2 SO_2/3_ multimer, before and after treatment. Although both nontreated OSA subgroups were quite different from the Snorer group, they were more dissimilar to each other than they were with the Snorer group. After treatment, both OSA subgroups tend to be closer to Snorer in terms of similarity, being the subgroup with PAP-induced PRDX2 SO_2/3_ multimer the most distant (dissimilar) to the Snorer group. The differences between these OSA subgroups diminished after treatment but only slightly. This data confirmed that these two OSA subgroups were heterogeneous in terms of clinical presentation, comorbidity, or risk factors. These data confirm our conviction that information on the redox status of enzymes such as PRDX2 and GAPDH in RBC might help to better recognize OSA subtypes and predict the therapeutic response in these patients.

Limitations in this study should be addressed. Sample size, female gender exclusion, the OSA group being constituted by non-diabetics but with higher levels of blood insulin and HOMA-IR, and the Snorer group being constituted by no completely healthy subjects limit the data generalization. Although patients were instructed to follow a restricted diet for three days before urine/blood collection to minimize its impact in patient’s antioxidant status and catecholamine determination, their dietary habits were not fully controlled. Proteomics and validation analysis were performed on −80 °C stored samples, which could introduce some bias in the molecular events of both control and disease samples.

## 5. Conclusions

The redox–oligomeric states of GAPDH and PRDX2 involving overoxidation by sulfinic/sulfonic acids were differentially modulated in OSA RBC probably due to the nocturnal apnea-induced intermittent hypoxia that was chronically experienced by these patients. This observation together with the extensive knowledge in the field point toward disturbance in GAPDH-dependent metabolic adaptation upon exposure to different O_2_ gradients along RBC circulation and decrease in PRDX2-mediated signaling and chaperone protection, which together strongly compromise antioxidant capacity of RBC to cope with oxidative stress.

PAP reoxygenation modulated redox–oligomeric states of GAPDH and PRDX2 toward their restoration, which might improve OSA compromised RBC homeostasis. PAP also induced the occurrence of PRDX2 sulfinylated/sulfonylated multimer in some patients along with an increase of GAPDH sulfonylated tetrameric/oligomeric forms. This phenomenon may be associated with “new gain” of function in GAPDH and PRDX2 such as eryptosis and chaperone protection, respectively. The complex interaction between pathophysiologic features of OSA and OSA metabolic comorbidities seemed to modulate the variability in GAPDH/PRDX2 redox–oligomeric responses following PAP treatment among patients.

Thus, information on the redox status of PRDX2 and GAPDH in RBC will help to better recognize OSA subtypes and predict the therapeutic response in these patients. Indeed, GAPDH combined with BMI and PRDX2 S-S Dimer combined with HOMA-IR showed to be very promising biomarkers to predict OSA and OSA severity, respectively.

A deeper understanding of the functional impact of PTMs on RBC GAPDH/PRDX2 regulation will be crucial to better understand RBC homeostasis in the context of OSA. The development of high throughput technologies other than WB will be necessary to validate in a large cohort of patients the value of GAPDH and PRDX2 as candidate biomarkers for OSA diagnosis and prognosis, respectively.

## Figures and Tables

**Table 1:** Cohort I—Discovery phase.

Demographic, Polysomnographic, and Analytical Characterization					
Demographic and PSG Parameters	Screened Subjects				
	Mean (Standard Deviation)			*p* Value *(*<0.05)	
	Snorers (*n* = 10)	OSA (*n* = 10)	PAP (*n* = 10)	Snorers vs. OSA	OSA vs. PAP
Age (years)	45.6 (10.9)	46.4 (6.5)	-	NS	*n*/a
**Habits**					
Current Smoking (*n*)	2	1	-	-	-
EPW Score	9.7 (6.5)	7.7 (2.9)	4.7 (3.8)	NS	NS
**Observational features**					
Morning arterial pressure (mmHg) *	132.5 (17.0)/80.9 (11.0)	138.4 (12.6)/90.7 (10.4)	-	*n*/a	*n*/a
BMI (kg/m^2^)	26.7 (1.6)	30.1 (2.9)	-	0.006	*n*/a
Abdominal perimeter (cm)	95.5 (4.4)	106.1 (10.4)	-	0.012	*n*/a
**Comorbidities**					
Hypertension (*n*)	3	7	-	-	-
Respiratory diseases (*n*)	0	0	-	-	-
Dyslipidemia (*n*)	3	6	-	-	-
Diabetes (*n*)	0	0	-	-	-
**Polysomnographic parameters**					
Mild/Moderate/Severe (*n*)	-	-/-/10	-	*n*/a	*n*/a
RDI (events/h)	3.1 (1.2)	53.7 (17.3)	-	0.011	*n*/a
ODI (events/h)	3.3 (4.7)	46.2 (22.9)	-	<0.001	*n*/a
Sleep efficiency (%)	78.4 (15.2)	73.6 (12.7)	-	*n*/a	*n*/a
Arousal index (%)	14.7 (6.7)	38.2 (15.3)	-	<0.001	*n*/a
Minimum Arterial Saturation (%)	89.3 (0.03)	78.2 (0.08)	-	<0.001	*n*/a
**PAP record**					
Number of days without use	-	-	53.2 (64.9)	-	-
Total of recording days	-	-	291.6 (158.7)	-	-
Residual AHI	-	-	1.9 (1.5)	-	-
**Analytical parameters**					
**Glycemic profile**					
Glucose (70–110 mg/dL)	96.7 (9.4)	96.2 (11.5)	92.9 (14.9)	NS	NS
HbA1C (4–6%)	5.7 (0.3)	5.8 (0.4)	5.6 (0.3)	NS	0.027
Insulin (3–25 mU/L)	13.1 (6.7)	23.6 (13.5)	41.1 (43.3)	0.047	NS
HOMA-IR (<2.15)	3.1 (1.6)	5.7 (3.2)	9.9 (11.2)	0.041	NS
**Lipid profile**					
Cholesterol (<190 mg/dL)	202.8 (37.1)	189.7 (29.9)	177.2 (35.6	NS	NS
Triglycerides (<150 mg/dL)	127.3 (66.7)	155.6 (92.2)	141.4 (75.2)	NS	NS
**Cardiovascular marker**					
Homocysteine (3.7–13.9 µmol/L)	14.8 (3.0)	16.0 (3.4)	16.6 (3.3)	NS	NS
**Urinary catecholamines**					
Adrenaline (1.7–22.4 µg/24h)	20.9 (13.7)	61.2 (142.8)	15.3 (7.3)	NS	NS
Nor-adrenaline (12.1–85.5 µg/24 h)	54.6 (16.5)	252.9 (565.5)	62.5 (25.5)	NS	NS
Dopamine (0–498 μg/24 h)	297.3 (94.9)	1066.9 (2403.7)	372.4 (146.9)	NS	NS
**Complete Hemogram**					
RBC (4.5–5.9 × 10^12^/L)	5.1 (0.3)	5.3 (0.3)	5.2 (0.3)	NS	NS
Hemoglobin (13–17.5 g/dL)	15.3 (0.8)	16.0 (0.9)	15.4 (1.1)	NS	0.007
Hematocrit (40–50%)	45.1 (2.9)	47.0 (3.01)	45.9 (2.8)	NS	NS
MCV (80–97 fL)	89.3 (4.7)	89.1 (4.7)	88.8 (4.9)	NS	NS
RDW (11.5–14.5%)	13.4 (0.6)	13.9 (0.8)	13.7 (0.6)	NS	NS
Platelets (150–450 × 10^3^µL)	230.7 (33.6)	217.0 (48.9)	194.1 (46.5)	NS	NS

NS: non-statistical meaning; *n*/a: not-applicable; * PA max/PA min.

**Table 2:** Cohort II—Validation phase.

Demographic. Polysomnographic and Analytical Characterization					
Demographic and PSG Parameters	Screened Subjects				
	Mean (Standard Deviation)			*p* Value (<0.05)	
	Snorers (*n* = 23)	OSA (*n* = 36)	PAP (*n* = 36)	Snorers vs. OSA	OSA vs. PAP
Age (years)	44.8 (9.6)	47.1 (7.5)	-	NS	*n*/a
**Habits**					
Current Smoking (*n*)	9	6	-	NS	*n*/a
EPW Score	9.5 (4.8)	10.8 (4.9)	6.2 (4.4)	NS	<0.001
**Observational features**					
Morning arterial pressure (mmHg) *	134.9 (17.2)/83.4 (11.9)	131.0 (16.9)/83.7 (11.9)	-	*n*/a	*n*/a
BMI (kg/m^2^)	27.2 (3.2)	30.2 (2.9)	-	<0.001	*n*/a
Abdominal perimeter (cm)	97.5 (7.7)	106.0 (8.1)	-	<0.001	*n*/a
**Comorbidities**					
Hypertension (*n*)	6	23	-	-	-
Respiratory diseases (*n*)	0	0	-	-	-
Dyslipidemia (*n*)	9	18	-	-	-
Diabetes (*n*)	0	0	-	-	-
**Polysomnographic parameters**					
Mild/Moderate/Severe (*n*)	-	16/3/17	-	*n*/a	*n*/a
RDI (events/h)	2.7 (1.4)	31.7 (25.2)	-	<0.001	*n*/a
ODI (events/h)	2.2 (3.3)	26.3 (25.4)	-	<0.001	*n*/a
Sleep efficiency (%)	78.1 (12.2)	74.6 (16.8)	-	NS	*n*/a
Arousal index (%)	14.2 (6.0)	28.3 (17.8)	-	<0.001	*n*/a
Minimum Arterial Saturation (%)	89.3 (2.9)	82.6 (6.3)	-	<0.001	*n*/a
**PAP record**					
Number of days without use	-	-	42.8 (47.5)	-	-
Total of recording days	-	-	275.6 (118.2)	-	-
Residual AHI	-	-	1.7 (1.2)	-	-
**Analytical parameters**					
**Glycemic profile**					
Glucose (70–110 mg/dL)	92.9 (7.9)	95.7 (12.2)	93.9 (13.7)	NS	NS
HbA1C (4–6%)	5.5 (0.4)	5.6 (0.4)	5.6 (0.6)	NS	NS
Insulin (3–25 mU/L)	12.4 (6.1)	16.4 (10.1)	24.6 (31.0)	NS	NS
HOMA-IR (<2.15)	2.9 (1.5)	3.9 (2.6)	6.2 (8.4)	0.040	NS
**Lipid profile**					
Cholesterol (<190 mg/dL)	190.3 (37.0)	186.7 (39.5)	182.3 (31.6)	NS	NS
Triglycerides (<150 mg/dL)	118.9 (62.9)	134.1 (66.1)	139.6 (83.4)	NS	NS
**Cardiovascular marker**					
Homocysteine (3.7–13.9 µmol/L)	15.4 (3.8)	16.2 (6.7)	17.1 (5.6)	NS	NS
**Urinary catecholamines**					
Adrenaline (1.7–22.4 µg/24 h)	20.4 (17.7)	28.6 (76.1)	17.2 (9.4)	NS	NS
Nor-adrenaline (12.1–85.5 µg/24 h)	64.0 (29.4)	117.9 (300.5)	55.0 (20.9)	NS	NS
Dopamine (0–498 μg/24 h)	375.5 (201.0)	547.1 (1273.6)	313.8 (133.0)	NS	NS
**Complete Hemogram**					
RBC (4.5–5.9 × 10^12^/L)	5.1 (0.4)	5.1 (0.3)	5.0 (0.3)	NS	<0.001
Hemoglobin (13–17.5 g/dL)	15.3 (0.8)	15.6 (1.0)	15.1 (0.9)	NS	<0.001
Hematocrit (40–50%)	45.0 (2.1)	45.6 (2.9)	44.3 (2.7)	NS	<0.001
MCV (80–97 fL)	89.1 (5.6)	88.6 (3.8)	88.5 (3.4)	NS	NS
RDW (11.5–14.5%)	13.5 (0.5)	13.4 (0.7)	13.7 (0.6)	NS	NS
Platelets (150–450 × 10^3^µL)	232.2 (47.5)	226.2 (48.6)	204.6 (46.4)	NS	<0.001

NS: non-statistical meaning; *n*/a: not-applicable; * PA max/PA min.

**Table 3:** Correlation between peroxiredoxin-2 (PRDX2) and GAPDH redox–oligoforms in OSA before (t0) and after six months of PAP treatment (t6).

	GAPDH Correlate		OSA		PAP	
			Pearson *r* Value	*p* Value	Pearson *r* Value	*p* Value
**PRDX2**	**S-S/S-S Dimer**	Tetramer	−0.512 *	0.025	-	-
		SO_3_ Tetramer	−0.483 *	0.036	-	-
		SO_3_ Oligomers	−0.473 *	0.041	-	-
	**SO_2/3_ Monomer**	Monomer	-	-	−0.551 *	0.015
		Tetramer	-	-	−0.551 *	0.015
		Oligomers	-	-	−0.485 *	0.035
		SO_3_ Tetramer	-	-	−0.506 *	0.027
		SO_3_ Oligomers	-	-	−0.516 *	0.024
	**SO_2/3_ Multimer**	Monomer	-	-	0.526 *	0.021
		Tetramer	-	-	0.777 ***	<0.001
		Oligomers	-	-	0.712 ***	0.001
		SO_3_ Tetramer	-	-	0.838 ***	<0.001
		SO_3_ Oligomers	-	-	0.787 ***	<0.001

* Correlation is significant at the 0.05 level (2-tailed). *** Correlation is significant at the 0.001 level (2-tailed).

**Table 4:** Correlation of the different redox/oligomeric states of GAPDH and PRDX2 with the study clinical parameters in OSA patients before and after PAP treatment.

Protein	Oligomers	Correlate	OSA		PAP	
			Pearson *r* Value	*p* Value	Pearson *r* Value	*p* Value
**GAPDH**	**Monomer**	RBC	0.389 *	0.019	-	-
		Hb	0.392 *	0.018	-	-
		RDW	-	-	0.363 *	0.029
		RDI	0.375 *	0.024	-	-
	**Tetramer**	HbA1C	-	-	0.336 *	0.045
		MCV	−0.359 *	0.032	-	-
		ADR	-	-	0.490 **	0.002
	**Oligomers**	HbA1C	−0.337 *	0.044	-	-
		MCV	−0.339 *	0.043	-	-
		ADR	-	-	0.436 **	0.008
		EPW	-	-	0.335 *	0.046
	**SO_3_ Tetramer**	HbA1C	−0.359 *	0.031	0.421 *	0.010
		TG	-	-	0.341 *	0.042
		ADR	-	-	0.553 ***	<0.001
		HCY	-	-	0.355 *	0.034
	**SO_3_ Oligomers**	HbA1C	−0.354 *	0.034	0.362 *	0.030
		ADR	-	-	0.479 **	0.003
**PRDX2**	**Monomer**	TG	−0.593 **	0.007	-	-
		EPW	-	-	0.557 *	0.013
		HCY	0.469 *	0.043	-	-
	**S-S/S-S Dimer**	RDW	−0.577 **	0.010	-	-
		PLT	0.510 *	0.026	-	-
	**S-S Dimer**	INS	−0.462 *	0.047	-	-
		HOMA-IR	−0.476 *	0.040	-	-
		RDW	−0.457 *	0.049	-	-
		PLT	0.552 *	0.014	-	-
		EPW	0.523 *	0.022	-	-
		RDI	−0.570*	0.011	-	-
	**SO_2/3_ Monomer**	GLC	-	-	−0.601 **	0.007
		ADR	-	-	−0.456 *	0.050
	**S-S/SO_2/3_ Dimer**	HbA1C	-	-	0.549 *	0.015
		RDW	−0.465 *	0.045	-	-
		PLT	0.508 *	0.026	-	-
	**SO_2/3_ Multimer**	TG	-	-	0.479 *	0.038
		ADR	-	-	0.772 ***	<0.001

* Correlation is significant at the 0.05 level (2-tailed). ** Correlation is significant at the 0.01 level (2-tailed). *** Correlation is significant at the 0.001 level (2-tailed).

## Supplementary Materials

The following are available online at https://www.mdpi.com/2076-3921/9/12/1184/s1, Table S1: Cohort III Validation phase. Table S2: List of identified RBC cytosolic proteins. Figure S1: Graphical representation of the 14 differentially abundant spot-proteins among Snorer, OSA and OSA after PAP treatment.

## Abbreviations

ADR	Adrenaline
AHI	Apnea-hypopnea index
BMI	Body mass index
DA	Dopamine
EPW	Epworth sleepiness scale
GAPDH	Glyceraldehyde-3-phosphate dehydrogenase
GLC	Glucose
Hb	Hemoglobin
HbA1C	Hemoglobin glycated
HCT	Hematocrit
HCY	Homocysteine
HOMA-IR	Model assessment of insulin resistance
INS	Insulin
MCV	Mean corpuscular volume
NAd	Nor-Adrenaline
ODI	Oxygen desaturation index
OSA	Obstructive sleep apnea
PAP	Positive airway pressure
PLT	Platelets
PRDX2	Peroxiredoxin-2
RBC	Red blood cell(s)
RDI	Respiratory disturbance index
RDW	Red cell distribution width
TC	Total cholesterol
TG	Triglycerides
t0	Before PAP treatment
t6	After six month of PAP treatment
