# Supplementary material for: Redox–Oligomeric State of Peroxiredoxin-2 and Glyceraldehyde-3-Phosphate Dehydrogenase in Obstructive Sleep Apnea Red Blood Cells under Positive Airway Pressure Therapy
Source: Antioxidants (Basel). 2020 Nov 26;9(12):1184. doi: 10.3390/antiox9121184 (PMC7761104; doi:10.3390/antiox9121184)
Supplement: Supplementary file 1 [file antioxidants-09-01184-s001.pdf]

Table 1. Cohort III – Validation phase.

Demographic, Polysomnographic and Analytical Characterization					
**Demographic and PSG parameters**	**Screened Subjects Mean (Standard Deviation)**			***p* Value (< 0.05)**	
	**Snorers (*n* = 18)**	**OSA (*n* = 19)**	**PAP (*n* = 19)**	**Snorers *vs.* OSA**	**OSA *vs.* PAP**
Age (years)	45.2	45.7	-	NS	n/a
**Habits**					
Current Smoking (*n*)	6	2	-	NS	n/a
EPW Score	10.1 (5.1)	9.9 (4.1)	5.9 (4.2)	NS	<0.001
**Observational features**					
Morning arterial pressure (mmHg)*	134.5 (17.2) / 82.4 (11.3)	130.5 (17.4) / 84.6 (14.4)	-	n/a	n/a
BMI (kg/m^2)	27.1 (3.1)	30.4 (2.9)	-	0.002	n/a
Abdominal perimeter (cm)	96.8 (7.5)	106.6 (9.2)	-	0.001	n/a
**Comorbidities**					
Hypertension (*n*)	5	11	-	-	-
Respiratory diseases (*n*)	0	0	-	-	-
Dyslipidemia (*n*)	7	8	-	-	-
Diabetes (*n*)	0	0	-	-	-
**Polysomnographic parameters**					
Mild / Moderate / Severe (*n*)	-	4 / 2 / 13	-	n/a	n/a
RDI (events/h)	3.1 (1.2)	45.1 (25.8)	-	<0.001	n/a
ODI (events/h)	2.7 (3.6)	39.7 (27.6)	-	<0.001	n/a
Sleep efficiency (%)	78.1 (12.5)	76.2 (16.7)	-	NS	n/a
Arousal index (%)	14.6 (6.1)	34.9 (21.0)	-	<0.001	n/a
Minimum Arterial Saturation (%)	89.4 (2.5)	80.2 (7.0)	-	<0.001	n/a
**PAP record**					
Number of days without use	-	-	38.9 (50.6)	-	-
Total of recording days	-	-	304.8 (120.4)	-	-
Residual AHI	-	-	1.9 (1.3)	-	-
**Analytical parameters**					
**Glycemic profile**					
Glucose (70-110 mg/dL)	94.4 (7.8)	96.0 (13.4)	95.9 (15.7)	NS	NS
HbA1C (4-6%)	5.6 (0.4)	5.7 (0.4)	5.7 (0.7)	NS	NS
Insulin (3-25 mU/L)	13.2 (6.6)	19.8 (12.2)	35.0 (39.9)	0.046	NS
HOMA-IR (< 2.15)	3.1 (1.6)	4.9 (3.0)	8.8 (10.8)	0.032	NS
**Lipid profile**					
Cholesterol (< 190 mg/dL)	193.7 (32.5)	184.8 (26.1)	180.8 (29.8)	NS	NS
Triglycerides (< 150 mg/dL)	120.7 (63.5)	145.7 (78.7)	157.4 (102.5)	NS	NS
**Cardiovascular marker**					
Homocysteine (3.7-13.9 µmol/L)	14.7 (3.7)	15.5 (2.9)	16.4 (2.8)	NS	NS

**Urinary catecholamines**					
Adrenaline (1.7-22.4 μg/24h)	22.2 (18.9)	37.6 (104.5)	19.5 (11.2)	NS	NS
Nor-adrenaline (12.1-85.5 μg/24h)	56.1 (19.4)	160.4 (412.6)	55.0 (23.0)	NS	NS
Dopamine (0–498 μg/24h)	335.0 (168.8)	695.3 (1748.2)	308.3 (151.8)	NS	NS
**Complete Hemogram**					
RBC (4.5-5.9 × 10^{12}/L)	5.0 (0.4)	5.2 (0.3)	5.0 (0.3)	NS	0.017
Hemoglobin (13-17.5 g/dL)	15.2 (0.8)	15.7 (1.2)	15.2 (1.1)	NS	0.003
Hematocrit (40-50 %)	45.0 (2.3)	46.0 (3.3)	44.7 (3.1)	NS	0.008
MCV (80-97 fL)	89.9 (5.3)	88.6 (3.7)	88.7 (3.8)	NS	NS
RDW (11.5-14.5 %)	13.5 (0.5)	13.5 (0.8)	13.8 (0.53)	NS	NS
Platelets (150-450 × 10^3μL)	234.8 (43.7)	229.7 (44.8)	202.5 (43.1)	NS	0.002

NS: non-statistical meaning ; n/a: not-applicable; *PA max/PA min

Table 2. List of identified RBC cytosolic proteins.

Protein Name	Acession ID	MW/pI	Mascot Score	Sequence Coverage (%)	Matches
40S ribosomal protein S6	A2A3R5	25067/11.13	46	40	15
60S acidic ribosomal protein P0 (Fragment)	F8VWV4	12298/9.34	44	45	9
Albumin (Fragment)	F6KPG5	68484/5.73	216	41	39
APOL1 protein	Q2KHQ6	43957/5.84	50	22	12
Bisphosphoglycerate mutase	P07738	30158/6.10	917	82	35
Catalase	P04040	59947/6.90	318	33	23
Catalase	P04040	59947/6.90	222	43	34
Catalase	P04040	59947/6.90	72	29	14
Catalase	P04040	59947/6.90	731	51	40
Catalase	P04040	59947/6.90	1290	66	50
CD8 antigen (Fragment)	A0N2Q4	21853/9.85	46	21	10
cDNA FLJ10013 fis, clone HEMBA1000369, highly similar to PRKCA-binding protein	B3KM22	46997/5.17	47	20	13
cDNA FLJ10334 fis, clone NT2RM2000649, highly similar to Homo sapiens KIAA0676 protein	B3KM54	50261/4.84	46	14	11
cDNA FLJ40459 fis, clone TESTI2041800, highly similar to BISPHOSPHOGLYCERATE MUTASE	B3KUR3	28146/5.59	281	46	16
cDNA FLJ40459 fis, clone TESTI2041800, highly similar to BISPHOSPHOGLYCERATE MUTASE	B3KUR3	28146/5.59	267	42	15
cDNA FLJ53818, highly similar to Nucleolar transcription factor 1*	B4DNQ1	77584/5.66	47	23	26
cDNA FLJ54111, highly similar to Serotransferrin	B4DI57	65334/6.95	73	19	15
cDNA FLJ55253, highly similar to Actin, cytoplasmic 1	B4DW52	38950/5.19	47	29	13
cDNA FLJ55365, highly similar to splicing factor, arginine/serine-rich14	B4DSQ4	96352/8.03	45	11	19

cDNA FLJ56812, highly similar to Solute carrier family 12 member 5	B7Z3I0	100597/6.88	43	13	22
cDNA FLJ60299, highly similar to Rab GDP dissociation inhibitor beta	B4DLV7	51577/8.37	64	40	21
cDNA FLJ60299, highly similar to Rab GDP dissociation inhibitor beta	B4DLV7	51577/8.37	204	40	18
cDNA FLJ60461, highly similar to Peroxiredoxin-2*	B4DF70	20209/8.90	198	48	14
cDNA FLJ77404, highly similar to Homo sapiens small nuclear ribonucleoprotein 70kDa polypeptide	A8KAQ5	51522/10.01	50	30	19
cDNA, FLJ79229, highly similar to Lactotransferrin	B7ZAL5	74834/8.10	43	21	16
Coiled-coil domain-containing protein 84	Q86UT8	38463/8.48	53	23	14
Cytochrome P-450 2C (Fragment)	Q9UEH3	31622/8.99	56	26	13
Delta-aminolevulinic acid dehydratase	P13716	36728/6.32	327	55	28
Delta-aminolevulinic acid dehydratase	P13716	36728/6.32	294	47	27
Flavin reductase (NADPH)	P30043	22219/7.13	62	50	15
Flavin reductase (NADPH)	P30043	22219/7.13	57	60	11
Gamma-glutamyltransferase (Fragment)	Q9UEN8	5293/6.78	51	88	6
Glyceraldehyde-3-phosphate dehydrogenase*	E7EUT5	28024/6.45	184	63	15
Glyceraldehyde-3-phosphate dehydrogenase*	E7EUT5	28024/6.45	251	55	22
Glyceraldehyde-3-phosphate dehydrogenase OS*	P04406	36201/8.57	59	21	8
Glycophorin A	K9JHF1	1863/8.25	10	75	2
GMP reductase*	H0YLV5	36342/7.06	40	23	9
GRIP and coiled-coil domain-containing protein 2 (Fragment)	H7C010	10057/9.40	5	13	1
Hemoglobin alpha 1 globin chain (Fragment)*	Q9BX83	10703/7.06	125	69	6
Hemoglobin subunit alpha	P69905	15305/8.72	413	73	18
Hemoglobin subunit beta	P68871	16102/6.74	622	63	26
Hemoglobin subunit beta*	P68871	16102/6.74	401	83	16
Hemoglobin subunit beta*	P68871	16102/6.74	289	73	21
Hemoglobin subunit beta	P68871	16102/6.74	330	95	18
Hemoglobin subunit beta	P68871	16102/6.74	88	76	11
Hepcidin*	P81172	9915/9.24	5	7	1
Histone H1.3	P16402	22336/11.02	50	48	16
Insulin-like growth factor 2 mRNA-binding protein 3	O00425	64008/8.99	26	9	10
Kinesin family member 13B, isoform CRA_a	D3DSU3	175946/5.65	44	9	22
LEM domain-containing protein 2	D6RBV0	12873/9.92	49	35	9
MNDA protein (Fragment)*	Q05CU9	35074/9.83	61	30	16
N6-adenosine-methyltransferase subunit METTL14	Q9HCE5	52688/5.89	45	17	15
NPM1 protein*	Q9BTI9	25147/4.72	49	25	9
Nuclear pore complex-interacting protein (Fragment)	A0A067ZXN4	28012/10.83	36	23	10
Orphan sodium- and chloride-dependent neurotransmitter transporter NTT5 (Fragment)	M0QZL4	4034/7.90	7	15	1
Peroxiredoxin-2	P32119	22049/5.66	1180	77	34
Peroxiredoxin-2	P32119	22049/5.66	370	79	32
Peroxiredoxin-2	P32119	22049/5.66	768	63	26
Peroxiredoxin-2	P32119	22049/5.66	673	67	25

Peroxiredoxin-2	P32119	22049/5.66	182	74	25
Pescadillo homolog (Fragment)	H7C267	22353/4.83	58	27	15
Potassium voltage-gated channel subfamily KQT member 2	A0A0G2JRN9	43314/10.03	49	23	12
Proteasome subunit alpha type-2	P25787	25996/6.91	134	47	15
Protein polybromo-1 (Fragment)	H0Y5B5	127011/6.51	47	15	25
PTCH protein +4' (Fragment)	Q3LFT2	18696/9.38	43	23	8
Purine nucleoside phosphorylase	Q8N7G1	32758/6.71	50	24	9
Purine nucleoside phosphorylase	P00491	32325/6.45	155	40	17
Purine nucleoside phosphorylase	Q8N7G1	32758/6.71	53	39	16
Ribosomal protein L18a homologue (Fragment)	Q15371	18153/8.97	51	42	16
RNA-binding motif protein, X chromosome	H3BUY5	28728/9.89	40	30	12
SEC14-like protein 2 (Fragment)	C9JTM4	14474/7.65	69	28	10
Something about silencing protein 10	Q9NQZ2	54639/5.50	54	16	17
Splicing factor, arginine/serine-rich 4 variant (Fragment)	Q53F45	44289/11.39	19	10	7
Superoxide dismutase [Cu-Zn]	P00441	16154/5.70	267	73	12
Superoxide dismutase [Cu-Zn]	P00441	16154/5.70	77	62	7
Survival of motor neuron-related-splicing factor 30*	O75940	26866/6.78	47	29	11
TBC1 domain family member 3H*	P0C7X1	62921/9.20	54	24	21
Titin (Fragment)	H7C1P9	109224/5.38	57	16	28
Titin (Fragment)	H7C1P9	109224/5.38	50	16	26
Titin (Fragment)	H7C1P9	109224/5.38	61	17	28
Transmembrane protein 143 (Fragment)	M0QZ02	15824/8.89	34	40	6
Ubiquitin carboxyl-terminal hydrolase 14 (Fragment)	J3QQT6	2231/11.17	9	80	1
Uncharacterized protein C6orf141	Q5SZD1	26737/8.14	47	40	12
Uncharacterized protein C6orf203	Q9P0P8	28038/9.31	52	28	13
YWHAE/FAM22A fusion protein (Fragment)	G9K388	41540/4.86	69	31	17

* Proteins differentially abundant among Snorers, OSA and OSA after PAP, fold change ≥ 1.2; Anova $p < 0.05$.

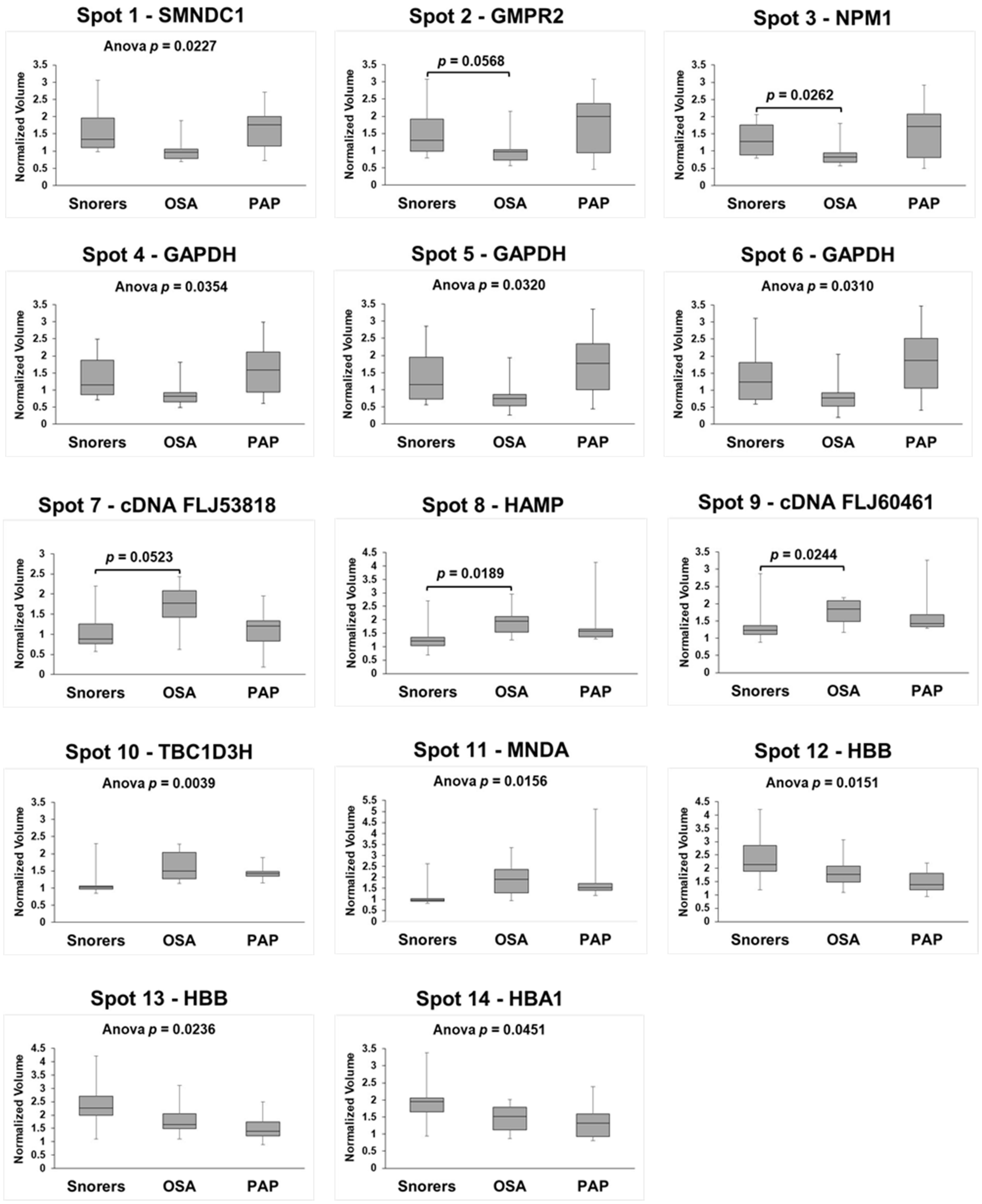

Figure S1. **Graphical representation of the 14 differentially abundant spot-proteins among Snorers, OSA and OSA after PAP treatment**. Graphical representations of the 14 protein spots detected by 2D-DIGE analysis (see Figure 1) and identified by imaging analysis using the Progenesis SameSpots, version 4.5 (Nonlinear Dynamics, Newcastle-Upon-Tyne, UK) as differentially abundant (fold change 1.2; Anova $p < 0.05$) among Snorers, OSA and OSA after PAP are shown. The identity (gene name) of each spot by mass spectrometry analysis are displayed, respectively.
